# Modeling and Analyzing Urban Sensor Network Connectivity Based on Open Data

**DOI:** 10.3390/s23239559

**Published:** 2023-12-01

**Authors:** Bartosz Musznicki, Maciej Piechowiak, Piotr Zwierzykowski

**Affiliations:** 1 Institute of Computer and Communication Networks, Faculty of Computing and Telecommunications, Poznań University of Technology, 60-965 Poznań, Polandpiotr.zwierzykowski@put.poznan.pl (P.Z.); 2 Department of Computer Science, Kazimierz Wielki University, 85-064 Bydgoszcz, Poland

**Keywords:** urban sensor networks, open data, graph modeling, connectivity analyzing

## Abstract

The optimization of network topology is crucial to achieve efficient data transmission in wireless sensor networks. Recently it has been proven that emerging open data sources can be used for modeling the structures of heterogeneous urban sensor networks. With this, leveraging real location data of various networked and sensing devices became feasible and essential. This approach enables the construction and analysis of more accurate representations based on frequently updated actual network infrastructure topology data, as opposed to using synthetic models or test environments. The presented modeling method serves as the basis for the designed architecture and implemented research environment. This paper introduces a set of algorithms which transform devices’ location data into graph-based wireless network connectivity models. Each algorithm is thoroughly discussed and evaluated. Moreover, static (momentary) and dynamic (time-spanning) network topologies are constructed in four large Polish cities based on publicly available data. Multidimensional simulation-based analysis is conducted to investigate the characteristics of the modeled structures. Directions for further research are suggested as well.

## 1. Introduction

*Wireless sensor networks* (WSNs) are usually imagined and designed as homogeneous structures, studied using synthetic models and computer simulations [1] or in experimental testbeds [2]. The research on some *vehicular ad hoc networks* (VANETs) and *delay-tolerant networks* (DTNs) was based on historical data obtained from transportation operators [3,4]. Another investigation used the readings gathered by a telecommunications operator in a proprietary urban mobile relay network [5]. In a world which is becoming increasingly networked, various new kinds of devices are connected in urban spaces, e.g., electricity meters, home automation and entertainment devices, trash bins, parking meters, etc. Some are designed specifically for sensing purposes while others are capable of performing different types of measurements in addition to their main functions. The boundaries between different types of hitherto studied networks are becoming blurred and their structures are becoming more heterogeneous.

**Figure 1:**
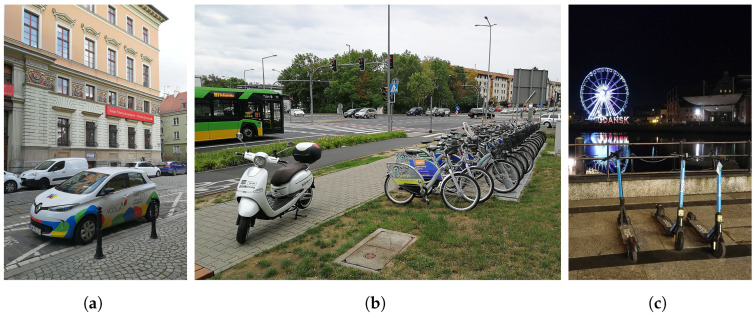
Examples of connected devices in cities in Poland. (**a**) Electric rental car and parking lot in Wrocław in December 2019; (**b**) bus, scooter, and bike rental station in Poznań in August 2019; (**c**) electric kick scooters in Gdańsk in November 2019.

Currently, new diverse online data sources are emerging. They include both ones that provide infrequently changing sets of data and ones that serve real-time data related to public transport vehicles and elements of urban infrastructure. More and more of these sources are available online and enable access to data related to, e.g., buses and trams, as well as public transport stops and ticket machines. Not only the geographic location of each element is available, but quite often also additional information, e.g., the type of the device, current running parameters, and recent values of the measurements. Due to the ongoing development of data storage, processing, and distribution technologies, these data can be made publicly available [6,7]. Therefore, further open data sources are expected to become available within the coming years. This opens up a range of unexploited research and development possibilities related to deterministically and randomly deployed nodes of sensing capabilities [8]. See the examples of such connected devices in cities in Poland in Figure 1.

This article builds on the original idea presented by the same team in [9] and extends it with a detailed study of the effectiveness of the proposed algorithms. It has been proven that open data can be used for modeling heterogeneous urban sensor networks. The actual types and features of these networks are reviewed and key routing research problems are defined. The characteristics of data sources are presented and different exemplary graphs are modeled to show the feasibility of the method and to indicate potential applications. Moreover, a practical network modeling architecture is introduced.

The next sections concretize and investigate the concept further. First, in Section 2, new urban sensor network connectivity modeling algorithms are presented and discussed. They include both static (momentary) and dynamic (time-spanning) graph-based network modeling methods. Then, Section 3 introduces the multidimensional simulation study’s methodology and architecture. Open data related to four Polish cities are used. Diverse geographic areas are defined and example modeled networks are presented. The results are thoroughly discussed in Section 4 and Section 5 to investigate the properties of each algorithm and modeled structure. Section 6 presents a summary of our findings.

## 2. Network Modeling Algorithms

**Figure 2:**
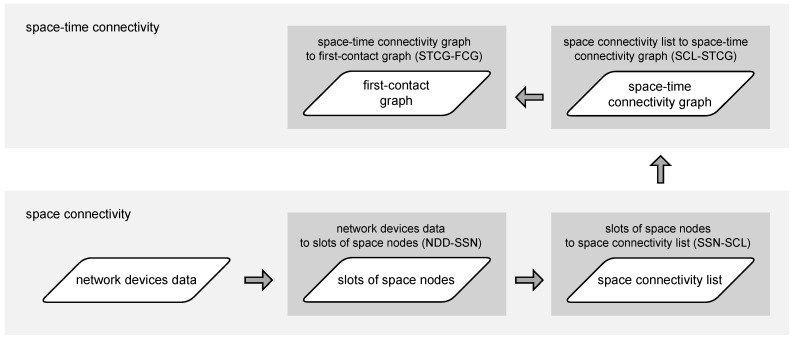
Network modeling flow.

The complete modeling flow, composed of algorithms introduced in the next subsections, is shown in Figure 2. Both static (space) and dynamic (space–time) realistic graphs can be generated to enable graph-based analysis of network topologies and routing algorithms of interest.

Time-changing graph representations and nomenclature were reviewed in [9] and can be referenced when needed. Based on the presented naming evolution, the terms *slot*, *space graph*, and *space–time graph*, as well as, e.g., *space edge* and *time edge*, will be used as the basis for naming modeled networks and their elements. Some graphs will be additionally termed *time-expanded* or *time-aggregated* to indicate if their form is layered or compacted.

The discussion of each algorithm is closed with a definition of time complexity. The usage of data structures implemented with hash tables is assumed and, therefore, average-case complexity O(1) applies for all basic data insertion, search, update, and deletion operations. These include, e.g., obtaining an element of a simple set, or accessing an element of a more advanced dictionary-like keyed structure. For this reason, the influence of this type of operation is not taken into account and the work centers on the presented time complexities in the very essence of the algorithms.

### 2.1. Network Device Data to Slots of Space Nodes

**Figure 3:**
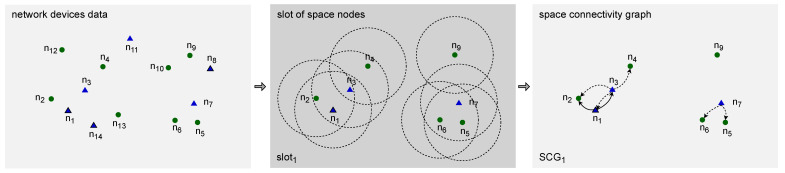
Space connectivity graph modeling example.

The first stage of modeling, as presented in Algorithm 1, *network device data to slots of space nodes* (NDD-SSN), is aimed at data quantization, i.e., the construction of a list of subsequent time slots. The term comes from the research of Huang et al., where it was used to denote the space between consecutive layers of a *space–time graph* [10]. In the presented novel network modeling approach, these slots are network topology snapshots that capture the deployment of modeled physical wireless network devices in consecutive intervals of slotLength. Each time slot groups the instances (occurrences) of all devices in a network area of interest considered to belong to a given timeFrame. Such a timeframe is defined for every network device class. The time distribution of data related to the devices is discrete. The two-dimensional area is defined by two space-related closed intervals. As a result, each actual device is represented as a node in the slots, and the device is considered to have occurred. The slots are sets of nodes only, i.e., the nodes are not connected with any edges yet, as presented in Figure 3. Stationary simple nodes are depicted with green circles, stationary advanced nodes are depicted with blue triangles, and mobile advanced nodes are blue triangles additionally marked with a black border.

**Algorithm 1:** Network device data to slots of space nodes.
**Input:**


$area\leftarrow (\left[X_{min},X_{max}\right],\left[Y_{min},Y_{max}\right])$

// (network area of interest(

$classes\leftarrow {\left(class_{i}\right)}_{i\leftarrow 1}^{j}:$

// (list of *j* distinct device classes(    $class_{i}\leftarrow {\left\{device_{k}\right\}}_{k\leftarrow 1}^{l}:$// (set of *l* devices of class *i*(      $device_{k},\leftarrow ($
// (device *k*(         $instances_{k},\leftarrow {\left(instance_{m}\right)}_{m\leftarrow 1}^{n}:$// (list of *n* time instances of device *k*(           $instance_{m}\leftarrow ($// (instance *m*(              $time_{m},$// (instance occurrence time(              $location_{m}$// (instance location(           ),
         $id_{k},$// (device id(         $role_{k}$// (device role(      )


$slotLength\in R_{>0}$

// (length of time slots(

$topologyLength\in N^{+}$

// (number of subsequent time slots of the time topology(

topologyStart

// (start time of the topology(

$windows\leftarrow {\left(window_{i}\right)}_{i\leftarrow 1}^{j}$

// (list of *j* time window lengths corresponding to the respective *j*(

device classes


**Output:**


$slots\leftarrow {\left(slot_{p}\right)}_{p\leftarrow 1}^{topologyLength}:$

// (list of subsequent time slots(    $slot_{p}\leftarrow {\left\{node_{r}\right\}}_{r\leftarrow 1}^{s}:$
// (set of all *s* nodes of slot *p*(      $node_{r}\leftarrow ($
// (node *r* of slot *p*(         $class_{r},$// (node class(         $id_{r},$// (node id(         $location_{r},$// (node geographic location(         $role_{r},$// (node role(         $slotNumber_{r}$// (node slot number(      )  **1** slots←assignDevicesInstancesToSlots()// (assign devices instances to slots of nodes(**2** **output** *slots*

The time topology begins at a given topologyStart time and its span, i.e., topologyLength is defined by the number of subsequent time slots of equal nonzero slotLength within the network modeling period. Each device in the area of interest is identified by a unique id, performs the desired network role, and belongs to one of the distinct classes. A data-lookup window of a given length, i.e., duration, is defined for each such class. Every time the occurrence of a device is distinguished is considered a time instance of this device. Every instance is marked with the occurrence time and location.

The Procedure *assignDevicesInstancesToSlots* starts with initializing the list of empty time slots, setting topologyStart as the initial slotEnd time and obtaining the number of device classes *j*. Then, it iteratively defines the sets of nodes that belong to each slot. Every iteration begins with moving the current value of slotEnd time by slotLength. Then, for each class, it is checked which time instance of every device in the network area is the newest time occurrence of that device in the timeFrame of interest. This timeFrame is defined as a half-open interval preceding slotEnd by the time length of the window of the class of the device. If newestInstance is selected, i.e., an instance which satisfies the time conditions related to current slot and class, a new node is added to the current slot. That node is marked with the class, id, location, slotNumber, and role of the instance of the given device. As a result, the list of slots of the nodes is obtained. It has to be pointed out that a list is an ordered sequence of elements while a set is an unordered collection.

Due to nested the iterative nature of the algorithm, its upper bound of time complexity is related to the number of slots (topology length), device classes, the number of the devices in the largest class, as well as the maximum number of device instances. Therefore, it can be defined as $O(|slots|\;\cdot \;|classes|\;\cdot \;|class{|}_{MAX}\;\cdot \;|instances{|}_{MAX})$.

**Procedure** assignDevicesInstancesToSlots

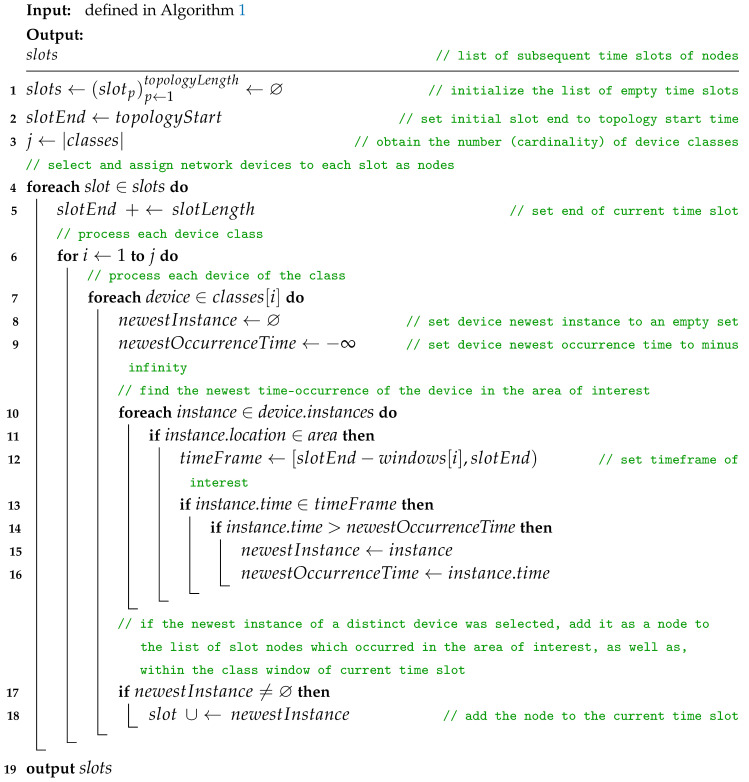



### 2.2. Slots of Space Nodes to Space Connectivity List

**Figure 4:**
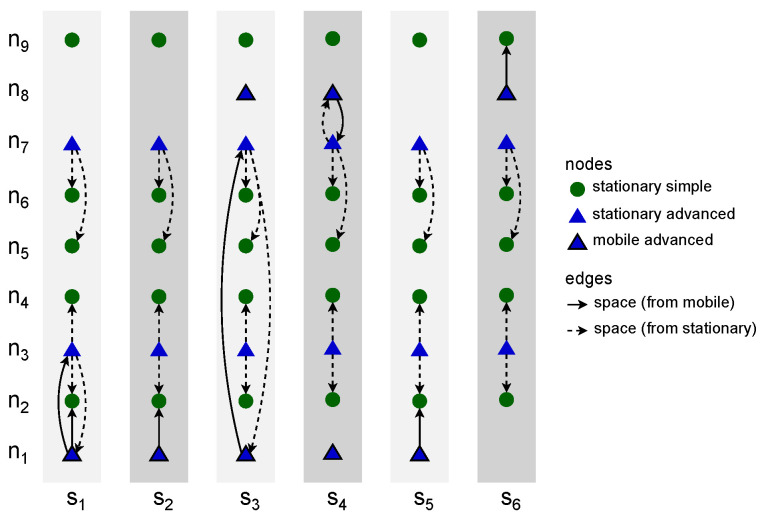
Example space connectivity list.

When the data related to the network devices have been turned into subsequent time slots of nodes, the next step of the modeling network topology can take place. Therefore, Algorithm 2, *slots of space nodes to space connectivity list* (SSN-SCL), constructs the *space connectivity list* (SCL), i.e., the list of subsequent time-ordered directed *space connectivity graphs* (SCGs)—based on the list of slots and the assumed radioCoverage of the devices. Such an SCL was called an *evolving graph* by Ferreira [11]. The graphs are considered to reflect possible temporary connectivity of the network in the related slot, as shown in Figure 3. Dashed links are the ones originating in stationary nodes, while solid links are those starting in mobile nodes. Here, radioCoverage can be a simple nonzero omnidirectional constant-range function, as well as an advanced model-based function which depends on, e.g., device class, state, radio transmission and reception capabilities, as well as propagation conditions. An example SCL with six slots and nine nodes is presented in Figure 4. It is assumed that nodes of a simple type can only be the ends of the edges (receivers), while advanced nodes can be also the start (transmitters). The number of nodes varies among the graphs and reflects the changes in the number of space nodes in the area of interest over time.

**Algorithm 2:** Slots of space nodes to space connectivity list

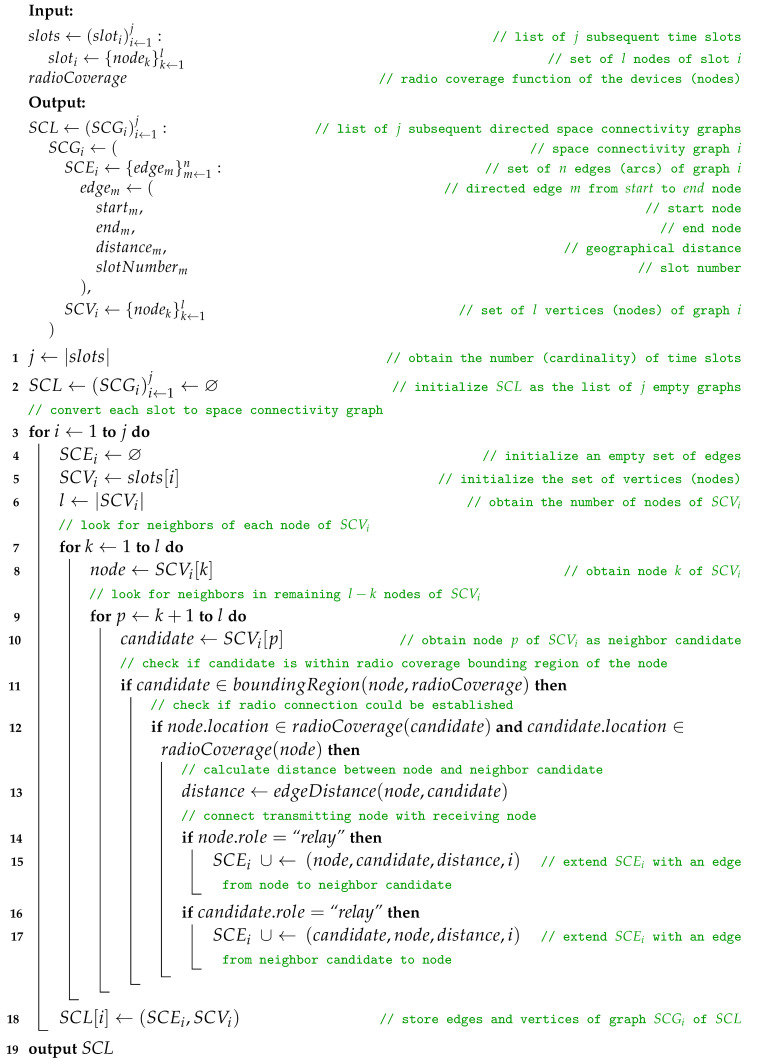



The boundingRegion is used to reduce the number of inter-nodal distance computations. This is based on the observation that for complex radioCoverage functions it is beneficial to perform the first rough neighbor candidate filtering in a less computationally intensive way. Afterwards, further precise calculations are performed only for the pairs of nodes that are close enough, and therefore, likely to be able to establish a connection—depending on the shape, size and center (location) of the radioCoverage of those nodes. The simplest approach to determine this, when working with a spheroid-based coordinate system, is to find a quasi-rectangular projected circumscribed area of the radioCoverage of the node. In the most simplistic case of a flat network area and uniform omnidirectional radioCoverage, the boundingRegion would be a circumscribed rectangle of a circle centered at the location of the node, with the radio range being the radius of this circle.

The algorithm begins by establishing the number *j* of time slots of the nodes, i.e., obtaining the cardinality of the list of slots, and initializes the list of *j* empty *SCG* graphs. Then, iteratively, each $SCG_{i}$ graph is filled with edges $SCE_{i}$ and vertices $SCV_{i}$. This list of *l* vertices is simply the list of all nodes of $slot_{i}$. The edges $SCE_{i}$ are determined in a more complex, and yet computational-complexity-optimized way.

For each $SCG_{i}$, an empty set of edges $SCE_{i}$ is initialized. Then, the set of vertices (nodes) $SCV_{i}$ is traversed. In every iteration, each not yet traversed vertex of $SCV_{i}$ is iteratively considered a neighbor candidate. By this means, the number of computations can be limited. In other words, the candidates are the l−k nodes which succeed the current node (the $k^{th}$ one) in the list of *l* nodes of $SCV_{i}$. This optimization can be applied because of the symmetric nature of the process and operations aimed both at the node and candidate can follow.

If the candidate is located within the boundingRegion of the node, then it is checked if the node is placed within the radioCoverage of the candidate, as well as the candidate being within radioCoverage of the node. When both conditions are met, the distance between the node and the candidate is calculated. The method edgeDistance can be a simple computation of geographical distance between two nodes by the means of determining the great-circle distance on a sphere [12]. It can be also a more complex metric function, e.g., related to the minimum power needed to complete a single transmission to the neighboring node. Next, if the node is a relay, then $E_{i}$ is extended with an edge from the node to a neighbor candidate. Similarly, an edge from a neighbor candidate to the node is added to the $SCE_{i}$ if the candidate is a relay. When all iterations are completed, the $SCE_{i}$ and $SCV_{i}$ of $SCG_{i}$ are stored in the SCL.

The radio-connectivity-related functions radioCoverage and boundingRegion are advised to be either precomputed for each node of the $SCV_{i}$ at the beginning of iteration *i*, or computed at the first usage and stored (cached) for future use, depending on the implementation. In the general urban use case, it can be assumed that the radioCoverage and network area dimensions would be of different orders of magnitude. Therefore, not using boundingRegion, especially with a large number of highly dispersed nodes, would lead to a significant increase in the computational complexity. In cases where the radioCoverage and dimensions of the network area tend to be of the same or similar orders of magnitude, especially in sparse networks, it might be beneficial to omit the computation and usage of boundingRegion. Similarly, the use of boundingRegion may be counter-effective if it is of a similar or higher computational complexity than checking if a node belongs to a region defined by radioCoverage.

The upper bound of the complexity of the algorithm is $O(|slots|\;\cdot \;{{\left|SCV\right|}_{MAX}}^{2})$, i.e., is related to the number of slots (topology length) and the number of nodes in a graph with the largest number of nodes of all graphs of the topology.

### 2.3. Space Connectivity List to Space–Time Connectivity Graph

**Figure 5:**
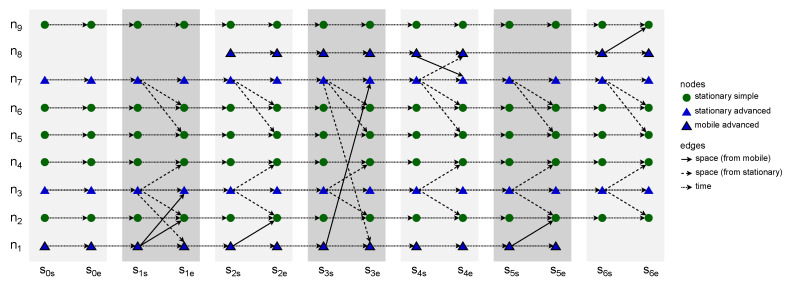
Example space–time connectivity graph.

The construction of a *space–time connectivity graph* (STCG) is defined as a more complex Algorithm 3, *space connectivity list to space–time connectivity graph* (SCL-STCG), composed of several procedures. Based on the structure of each directed space graph $SCG_{i}$ of SCL, time node instances as well as space–time edges are added to the STCG. These edges are of two types, i.e., *space*, and *time*, which indicates their role in the structure. Space edges connect different neighboring space nodes (devices) while time edges connect consecutive time instances of the same space node. Moreover, the notion of *intra-slot* and *inter-slot* edges is introduced to distinguish their graph roles. Intra-slot edges, that can be of *space* and *time* types, are used to construct the graph structure related to spatiotemporal relations within a given slot of nodes (based on $SCG_{i}$). Inter-slot edges are of the *time* type and connect slot-related structures (stages) to produce a space–time connectivity graph. An STCG is therefore a one-way multipath multistage layered structure that follows the direction of time and is a directed acyclic graph, as depicted in Figure 5.

**Algorithm 3:** Space connectivity list to space–time connectivity graph

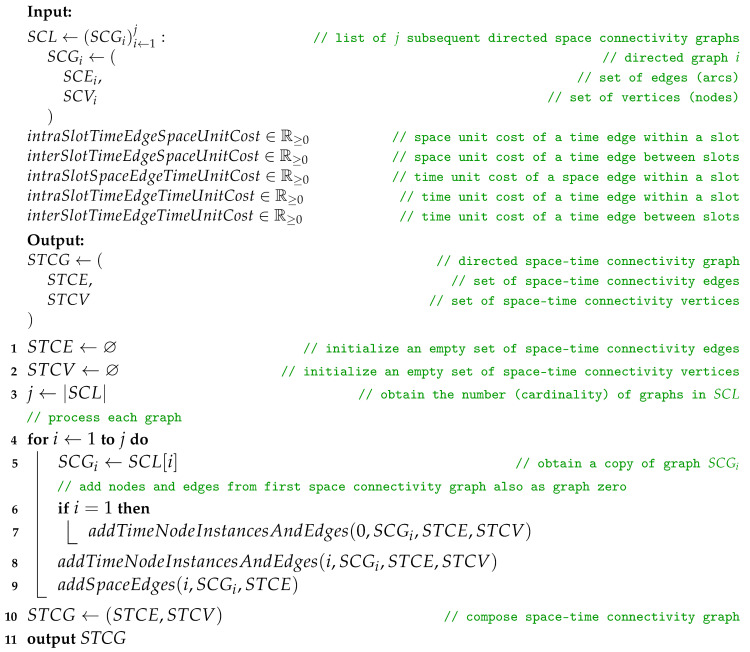



To enable the usage of various algorithms, such as the well-known ones related to finding shortest paths or trees, to process the constructed STCG as the input structure, the edges share the same set of attribute types, e.g., spaceDistance, and timeDistance. These weights are computed or set based on defined unit costs. They are a means of modifying or tweaking the resulting cost structure to meet the needs of further modeling and analysis:

- intraSlotTimeEdgeSpaceUnitCost—space unit cost of a time edge within a slot:
  − default value: 0;
  − meaning: nonzero value stands for unit space cost related to the node (device) operating within a time slot. It can be used, for example, to model the cost of receiving location beacons;
- interSlotTimeEdgeSpaceUnitCost—space unit cost of a time edge between slots:
  − default value: 0;
  − meaning: nonzero value stands for unit space cost related to the node (device) transitioning between time slots. It can be used, for example, to model the cost of transmitting location beacons;
- intraSlotSpaceEdgeTimeUnitCost—time unit cost of a space edge within a slot:
  − default value: 0;
  − meaning: nonzero value indicates unit time cost related to transmitting a message between two devices, e.g., due to technology-dependent buffering or delays. It can be used, for instance, together with intraSlotSpaceEdgeTimeUnitCost to favor intra-slot time edges over intra-slot space edges by path-finding algorithms. It will lead to maximizing buffering time in a single relay, minimizing the number of inter-node transmissions, and hence, the nodes involved. Although, it can happen at the expense of an overall increase in the space cost of the constructed STCG;
- intraSlotTimeEdgeTimeUnitCost—time unit cost of a time edge within a slot:
  − default value: 0;
  − meaning: should be considered in relation to intraSlotSpaceEdgeTimeUnitCost for given modeling scenario. It can also be used with interSlotTimeEdgeTimeUnitCost to shape time-path cost properties of STCG, e.g., as a tie-breaker;
- interSlotTimeEdgeTimeUnitCost—time unit cost of a time edge between slots:
  − default value: 1;
  − meaning: indicates unit cost related to transitioning (buffering) a message over time by a node. It is of key significance for path searching scenarios that aim to optimize the message delivery time, e.g., to minimize the total time cost of a path. If set to 0 it may lead to unexpected or erroneous results in optimization algorithms which are based on ordering the weights of the edges. It can be of use though when consciously used with properly selected values of intraSlotSpaceEdgeTimeUnitCost and intraSlotTimeEdgeTimeUnitCost.

Iterating over *SCL*, Procedure *addTimeNodeInstancesAndEdges* is invoked twice for $SCG_{1}$. The first, i.e., additional call, extends the sets of *space–time connectivity edges* (STCEs) and *space–time connectivity vertices* (STCVs) with time node instances and edges which represent the non-existent graph zero, as shown by Huang et al. [10]. Such an abstract graph $SCG_{0}$ with no space edges is required to provide correct starting points for path- and tree-finding algorithms and enable traversals based on the space and time metrics of the graph. For the remaining $SCG_{i}$ graphs, both space and time edges are constructed.

**Procedure** addTimeNodeInstancesAndEdges(i, SCG, STCE, STCV)

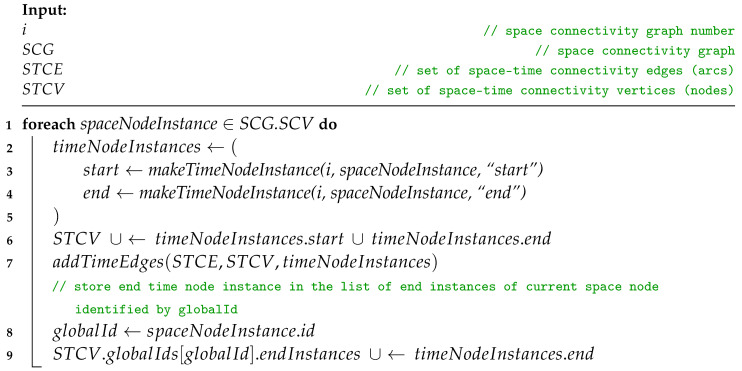



To add time node instances and edges in Procedure *addTimeNodeInstancesAndEdges*, each node of a given *graph* is used to make two new STCV nodes, which represent the instances of the node at the “*start*” and “*end*” of time slot *i*. The creation of these nodes is defined in Procedure *makeTimeNodeInstance*. To initialize a new timeNodeInstance, first the attributes of spaceNodeInstance are copied. Then, the id of the space node is stored as globalId to keep the reference of time node instance to its parent space node. Next, a new id is composed, i.e., the id of the space node is prefixed with slot number *i* and a time instance type indicator, either “*_s_*” for slot start, or “*_e_*” for slot end. In this way, for example, node “*1357*” of slot (space connectivity graph) number 3 will be converted to slot start instance “*3_s_1357*”.

**Procedure** makeTimeNodeInstance(i, spaceNodeInstance, timeInstanceType)

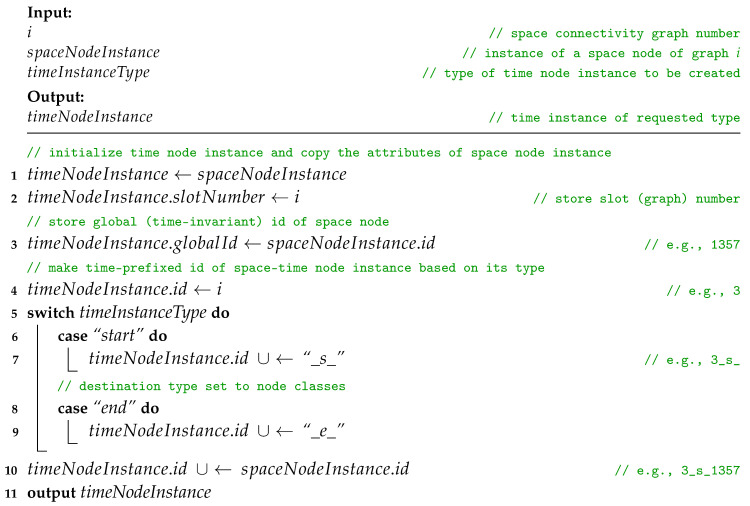



Afterwards, following Procedure *addTimeEdges*, *intraSlotTimeEdge* and *interSlotTimeEdge* are added to STCE. The directions are defined by the start and end node attributes and additional labels are set, i.e., spaceDistance, timeDistance, slotNumber, and type set to “*time*”. Here, timeDistance can mean, for example, the delay or buffering time related to traversing the edge by a message. Current slot node instances are connected with a *time* edge of intraSlotTimeEdgeSpaceUnitCost and intraSlotTimeEdgeTimeUnitCost. The current slot start instance is then linked with the newest slot end instance that exists in the set of *globalIds*, which is an attribute of STCV. This does not always mean it is connected with the end instance of the previous slot. The node instance might have not been present in the directly preceding slot, or the space node has not yet been present in the space–time graph. Then, the slot end instance is added to the list of endInstances of globalId in the globalIDs set related to the space nodes of STCV. Next, in Procedure *addSpaceEdges*, each space edge of $SCG_{i}$ is converted to *intraSlotSpaceEdge* and added to STCE. Finally, STCE and STCV are used to compose the STCG.

**Procedure** addTimeEdges(STCE, STCV, timeNodeInstances)

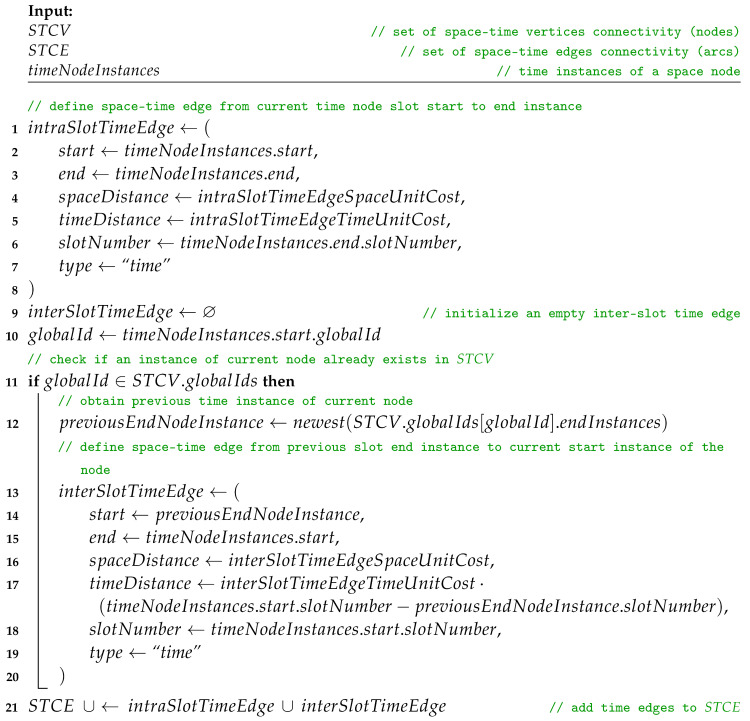



**Procedure** addSpaceEdges(i, SCG, STCE)

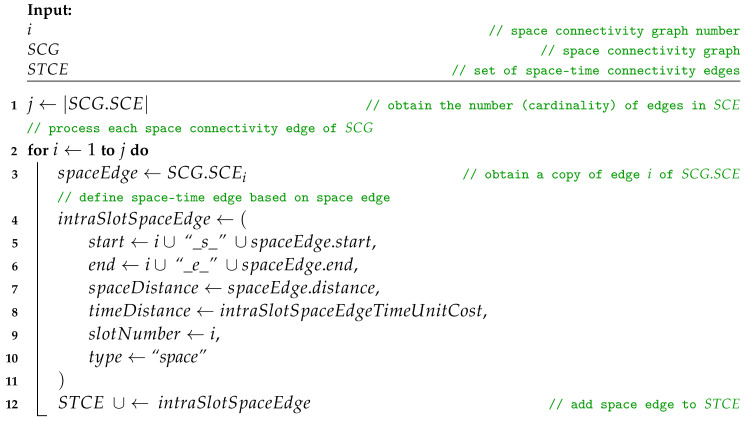



The upper bound of the time complexity of Algorithm 3 is $O(|SCL|\;\cdot \;{\left|SCV\right|}_{MAX}\;\cdot \;{\left|SCE\right|}_{MAX})$, and hence, is related to the number of space connectivity graphs, the number of nodes in the graph with the largest number of nodes, as well as the number of edges in the graph with the largest number of edges of all graphs of the topology.

*The space–time connectivity graph* (STCG) is an extension of the existing layered *space–time graph* (STG) concept [10,13]. It unambiguously reflects the space and time dimensions of changing network topology, and hence, enables multi-criteria spatiotemporal design and analysis. The essential innovations are the presented duplication of space nodes for each time slot as start and end node instances, as well as the introduction of intra-slot and inter-slot edges and metrics (e.g., the sets of $s_{1s}$ and $s_{1e}$ nodes and edges in Figure 5). They enable the development of new optimization algorithms and the proper usage of existing effective path-finding ones designed for graphs of more traditional time-flow-ignoring contexts, i.e., static as compared to dynamic (evolving) graphs. It is worth noting the term *time-expanded graph*, which was used in a related context [14]. In spite of that structure being an even more simplistic model, the term itself can additionally be of use in relation to space–time graphs because it captures and highlights the time-related graph structure span.

**Figure 6:**
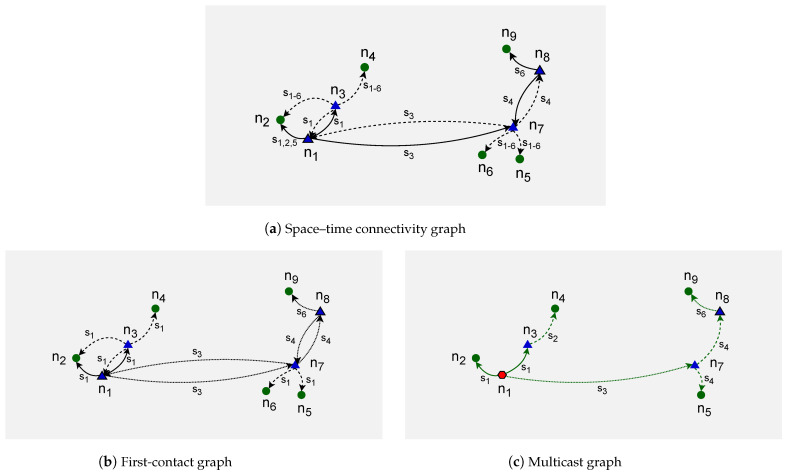
Example time-aggregated graphs.

In [14], the model of a *time-aggregated graph* is presented. This uses single instances of each node and edges between them. The edges are labeled with occurrence times of each connection. This alternative representation of a spatiotemporal graph, defined as a space connectivity list visible in Figure 4, is presented in Figure 6a. There, the directed edge label $s_{1,2,5}$ means that the link originated by a mobile node existed in time slots 1, 2, and 5. Similarly, $s_{1-6}$ denotes that the connection between stationary nodes that were present throughout the whole time span of modeled network.

Time-aggregated representation is not used to model STCGs because it does not enable direct use of well-known graph optimization and analysis algorithms which provide optimal solutions. However, methods are being developed that are aimed at solving these problems in *time-aggregated graphs*. The problem of determining minimum temporal paths is addressed by the algorithms for finding earliest-arrival, latest-departure, fastest, and shortest paths [15]. Methods for constructing a *directed Steiner tree* (DST) in a structure that resembles a *space–time graph* transformed from a *time-aggregated graph* called a *temporal graph* are also presented [16]. Similarly, in [10], they aim to construct a DST directly in the *space–time graph* used in their topology control efforts.

Importantly, a time-aggregated graph is a representation well-suited to capture the outcomes of algorithms that solve problems in STCGs. Therefore, it is used in the present research as a practical representation of the modeled first-contact and multicast graphs. Please see Figure 6b,c for examples.

### 2.4. Space–Time Connectivity Graph to First-Contact Graph

To build a *first-contact graph* (FCG), Algorithm 4, *space–time connectivity graph to first-contact graph* (STCG-FCG), is used. Such a graph is a *time-aggregated graph* with single instances of all spaceNodes located at the coordinates of their first instances, i.e., the ones in firstSlot (first *SCG*) in which the node was present. Each node is connected to each neighbor with a directed first-contact space or time edge. Space edges connect the nodes that are neighbors in the same firstSlot. Time edges connect them otherwise.

**Algorithm 4:** Space–time connectivity graph to first-contact graph **Input:** STCG←(// (directed space–time connectivity graph  STCE,// (set of space–time connectivity edges(  STCV// (set of space–time connectivity vertices( ) **Output:** FCG←(// (directed first-contact graph(  FCE,// (set of first-contact edges(  FCV// (set of first-contact vertices( )**1** spaceNodes←findFirstInstancesAndTimeNeighbors(STCG)**2** FCG←buildFirstContactGraph(STCG.STCV,spaceNodes)**3 output** *FCG*

**Figure 7:**
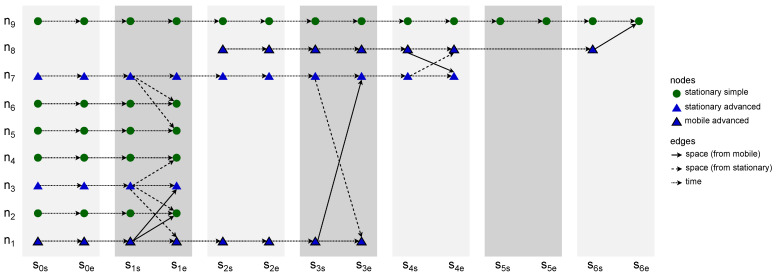
Example expanded first-contact graph.

**Figure 8:**
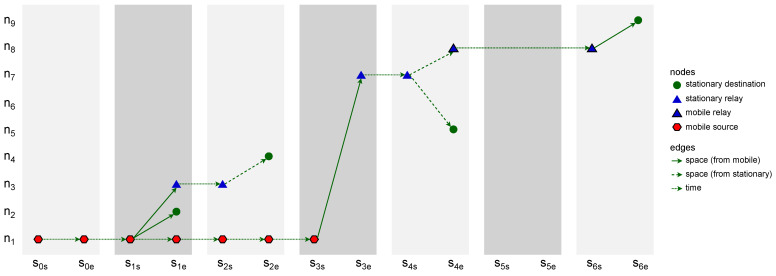
Example expanded space–time multicast graph.

An illustrative time-expanded FCG presented in Figure 7 was constructed in the STCG introduced in Figure 5. The related time-aggregated form is depicted in Figure 6b. The presented edge labels, for instance, $s_{3}$ and $s_{4}$, indicate in which time slot of a given slotNumber the firstContactEdge existed between two nodes. Similarly, an example space–time multicast graph was constructed and is presented in Figure 6c and Figure 8. It is a time-respecting tree that connects, over time, mobile source node $n_{1}$, via intermediate relay nodes, with four stationary destination nodes $n_{2}$, $n_{4}$, $n_{5}$, and $n_{9}$.

The STCG-FCG algorithm starts with finding first instances of space nodes and their first time neighbors in the STCG. The Procedure *findFirstInstancesAndTimeNeighbors* iterates over each nodeInstance of the STCG. At the beginning, it adds a new space node to the set of spaceNodes if it does not contain a node indexed with the globalId of the current nodeInstance. Key attributes of the new space node are inherited from the current nodeInstance, i.e., globalId becomes id, its id is set as firstInstance and its slotNumber becomes firstSlot. Moreover, an empty set of edges to successors is initialized. If a node indexed with globalId was present in spaceNodes, then firstInstance and firstSlot are updated if the slotNumber of the current nodeInstance is lower than firstSlot currently stored in spaceNodes for the current globalId of interest. This means, that current nodeInstance precedes the instance which has been so far considered the firstInstance (occurrence) of the given globalId (space node). The procedure closes with adding successors of current nodeInstance, which are instances of different space nodes. Its complexity is related to the number of nodes of the STCG, and hence, upper bounded by $O(|STCV{|}^{2})$.

**Procedure** findFirstInstancesAndTimeNeighbors(STCG)

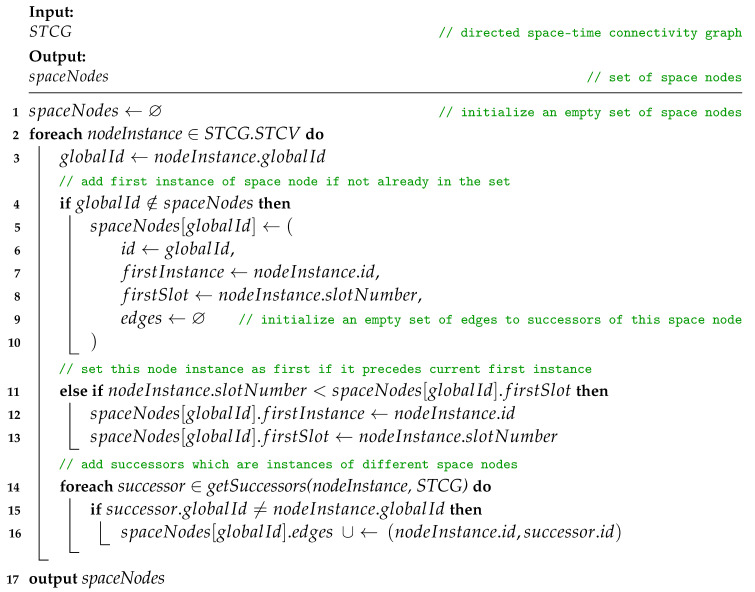



When the set of spaceNodes is ready, Procedure *buildFirstContactGraph* can be used to construct the FCG out of the STCG. At the beginning, all first node instances need to be added to the FCG. Therefore, for each spaceNode a firstContactNode is obtained. Such a node is the space node of the STCG, which was determined to be the firstInstance of a given spaceNode. Its id is set to the id of spaceNode, and then, the firstContactNode can be added to the set of vertices FCV of the FCG. In the next step, the edges going out from each firstContactNode are added to the FCG. For each node of the FCG, each edge going out from the current node is evaluated. The end node of the edge is set to be the current neighbor of the node. Then, the timeDistance between them is calculated and contactEdge defined as any edge connecting a node to a neighbor, based on their id and globalId, respectively. If such an edge does not exist in the FCG, then edgeType is set. It is “*time*” if timeDistance is positive and “*space*” otherwise. Then, firstContactEdge is defined and added to the FCG. If contactEdge exists in the FCG and its timeDistance is larger than timeDistance to the current neighbor instance, then the timeDistance of the edge of the FCG is updated to the shorter timeDistance.

As a result, FCG contains first instances of nodes labeled with space id. Those nodes are connected to their neighbors with first-contact edges. The upper bound of this procedure’s time complexity is $O(|spaceNodes{|}^{2})$.

**Procedure** buildFirstContactGraph(STCV, spaceNodes)

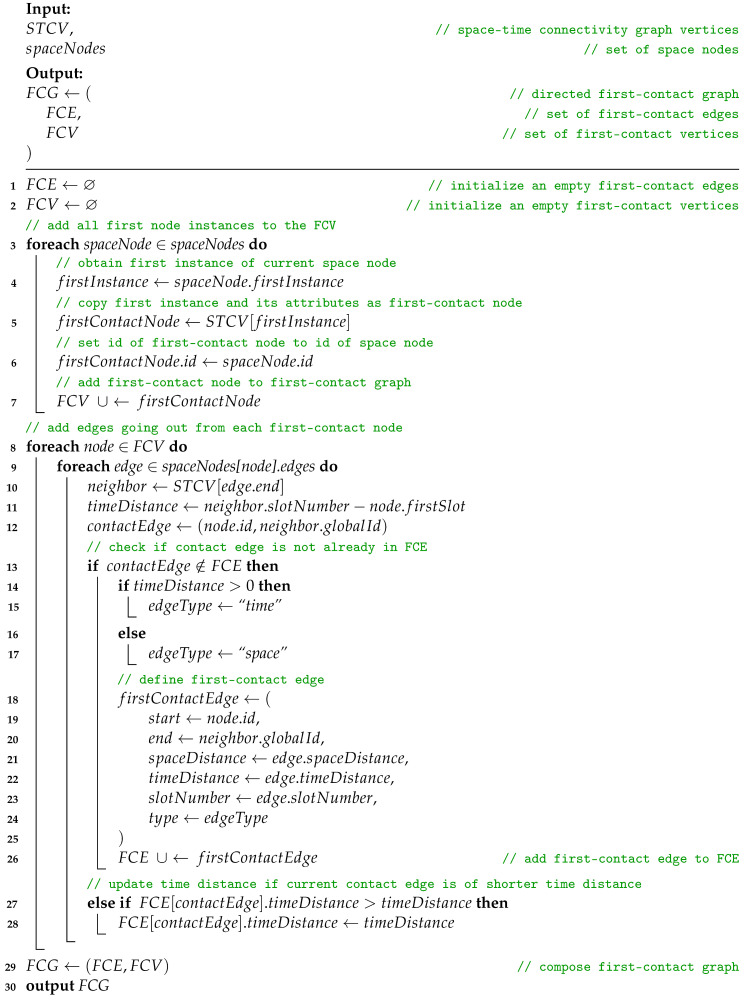



## 3. Simulation and Analysis Methodology

The main objective of the simulation study is to enable a multi-criteria evaluation and comparison of the proposed models and algorithms. Due to unknown characteristics of the underlying urban infrastructure, numerous features of the constructed networks are also of interest.

**Figure 9:**
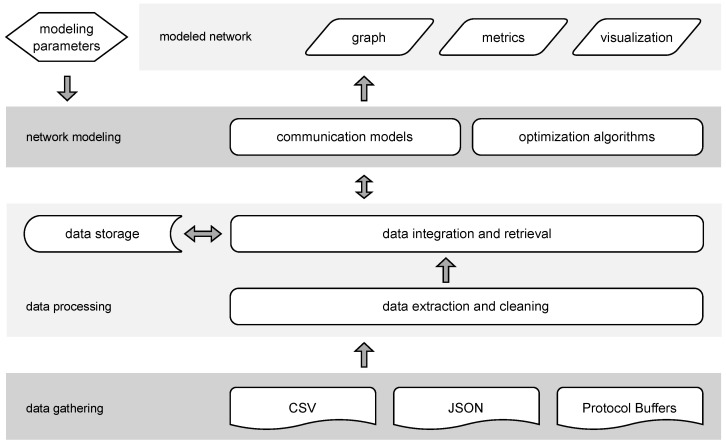
Open-data-based network modeling architecture [9].

The simulation environment was built as an extension of custom-made network modeling software [9] which implements the network modeling architecture presented in Figure 9. It is based on *Linux*, *PostgreSQL*, and *Python*, as well as on the *NetworkX* library that implements basic data structures and numerous standard graph-related operations [17]. The graphs are visualized using map data provided by *OpenStreetMap* [18]. The presented research environment has been implemented based on this software framework due to its ubiquity of use, detailed documentation, and broad community support. They provide numerous base functionalities used by graph researchers, hence have high popularity and proven value for data scientists.

**Figure 10:**
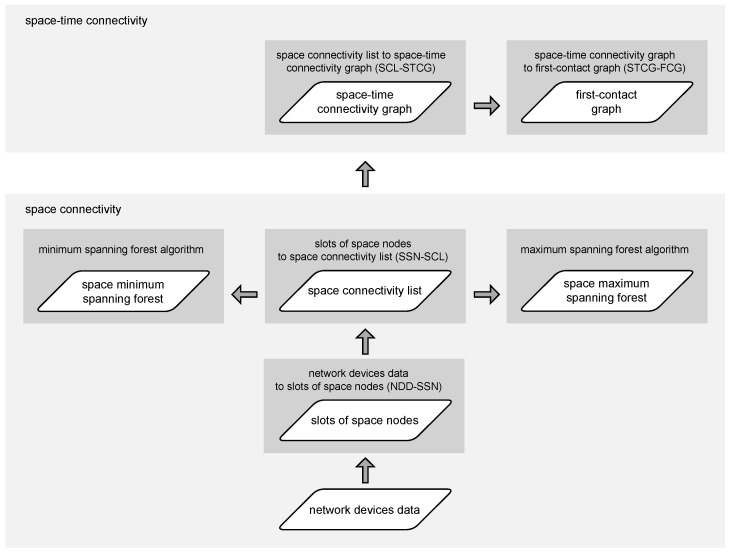
Simulation modeling flow.

The network modeling flow of the simulation follows a logical order in which the algorithms introduced in Section 2 are related to one another. The high-level steps of the simulation are depicted in Figure 10. The network topologies are constructed and analyzed as graphs. No actual radio propagation models, communication protocols, or power management mechanisms are simulated. By this means, technology and protocol agnosticism is ensured in all aspects. In this way, the efficacy and efficiency of the algorithms can be investigated and compared using graph theory methods. The key features and metrics of the modeled networks can be thoroughly analyzed as well.

### 3.1. Comparative Study Methodology

There exist no equivalent algorithms designed for modeling heterogeneous urban sensor networks. Therefore, the trends in the metrics of interest are compared and analyzed in relation to different urban areas, topology durations, and radio ranges. Space minimum and maximum spanning forests are constructed for each space connectivity graph undirected analog using Kruskal’s algorithm [19]. These forests are the generalized solutions of the problem of a *time-sub-interval minimum spanning tree* (TSMST) in a spatiotemporal network defined in [20]. Key metrics of the forests are compared to provide more insight into the momentary space connectivity topologies. Those forests are graphs composed of sets of trees built for every connected component of a space connectivity graph. The most informative node-related metrics of the constructed space–time connectivity graphs are compared to the related first-contact graphs. Further FCG-related parameters are gathered and investigated as well.

### 3.2. Statistical Analysis and Visualization

Statistical data are generated and gathered in the processes related to each step of the network modeling. Some of the metrics are calculated using custom-developed functions, while others are computed with the methods provided by the NetworkX graph modeling framework. The data are then represented as a pandas data structure called DataFrame [21]. In this way, advanced multidimensional data combining, filtering, categorization, and statistical processing is performed. Although, the values of individual data points and their numerical aggregates are not the center of attention in this study. The trends and relationships between the parameters of the networks and their metrics are the key concerns. Therefore, the analyzed data are visualized with the Seaborn data visualization library [22].

The data of interest are of a discrete nature, and therefore, the analyzed sets are presented in the form of scatter plots. The styles of the points represent different subsets of the data. Regression lines are overlaid on the plots to make the trends more visible in larger, denser, and overlapping data sets of a single chart, as in Figure 18. The sets of closely related subplots are grouped in named rows or columns of a single plot, e.g., the charts related to different graph types and radio ranges in Figure 23. Furthermore, pair plots are used to present the relationships between the sets of variables, as in Figure 26. A number of plots are categorical to group and shift the data horizontally around the values of interest, which makes the categories more distinguishable, such as city or knowledge mode. This kind of plot also introduces small jitter, i.e., random deviations, to horizontal distributions of the categories to make them more visible when there are multiple closely related values present. It does not change the values, i.e., the vertical distribution of the measurements, as visible in Figure 34. Due to the multidimensional nature of the data, the figures related to the subsets of metrics are also grouped and discussed in dedicated sections, e.g., in Section 4.1, focused on the space connectivity nodes’ parameters.

### 3.3. Simulation Data Sources and Node Classes

An investigation and analysis of the data sources discussed in [9] lead to the conclusion that sets of open data sources which meet the requirements of the study only exist for four Polish cities. Other cities provide limited scope or do not provide similar open data at all. The authors did not succeed in discovering equivalent open sets related to urban areas in other countries either. Therefore, Gdańsk, Poznań, Warsaw, and Wrocław, sources listed in Table 1, are used—being the ones that provide the data of comparable scope, granularity, and update frequency.

The geographic coordinates of the urban infrastructure elements extracted from the data are used in the presented comparative research and transformed using the introduced network modeling algorithms. In all four cities, the real-time locations of buses and trams are available. In Gdańsk and Poznań, the locations of public transport stops and ticket machines can be used. In Warsaw and Wrocław, ticket machines’ location data are not available. Although, in the case of Wrocław, the coordinates of city bike rental stations and parking lots (as shown in Figure 1a) of Vozilla (city electric car rental service) can be used instead. Most of the data are available in *JavaScript Object Notation* (JSON) format while the Poznań-related mobile nodes data are in *Protocol Buffers* (protobuf). The update frequencies of these data range from a few seconds in the cases of the continuously updated ones, to 10 and 20 s for Warsaw and Gdańsk, respectively. The numbers and locations of stationary nodes do not change that frequently. This means that even when the source updates the whole data set frequently, e.g., for air quality meters and city bike rental stations, the location-related data can remain unchanged for hours or days, just like for each 24 h of more infrequently updated ones. To enable heterogeneous structure modeling, the data sources were classified into three meaningful logical classes—mobile advanced, stationary advanced, and stationary simple. The nodes of each class are assigned a network role in the connectivity modeling scenarios:

- mobile advanced class ⇒ mobile relays;
- stationary advanced class ⇒ stationary relays;
- stationary simple class ⇒ stationary destinations.

It is assumed that advanced nodes are the nodes with more significant computing, storage, communications, and power resources. Therefore, they are capable of performing complex *delay-tolerant network* (DTN)-forwarding operations. In contrast, simple nodes are the simple recipients of the communication.

### 3.4. Simulation Areas and Example Modeled Networks

The four cities of interest—Gdańsk, Poznań, Warsaw, and Wrocław—are among the largest and the most populated ones in Poland, as presented in the next paragraphs. Warsaw is the most populated urban area, more than two and a half times the population of Wrocław. Wrocław is twenty three percent more populated than Poznań, while the population of Poznań is thirteen percent larger than the one of Gdańsk. The population densities also differ in a related way—Gdańsk is the least densely populated urban area, followed by Poznań, Wrocław, and Warsaw, which is almost two times more densely populated than Gdańsk. Interestingly, in terms of the expected numbers of public transport routes (lines) that operate during the day, Poznań is the city with the lowest number, followed by slightly more routes in Gdańsk and Wrocław. In Warsaw, twice as many routes are present on average.

An area of 3 by 2 kilometers was selected in each of the cities. This choice was aimed at covering partially alike and partially distinct regions that include both the busy heart as well as less dense surroundings of each urban area. A closer examination of the city topologies, visible in the presented figures, reveals unique terrain, building, street, and infrastructure layouts. Therefore, the modeled networks are expected to indicate both different and similar features.

Example presented graphs constructed in those areas show the state modeled on Wednesday, 27 November 2019, at 3:00 p.m., when omnidirectional radio coverage is assumed. The relays are dark blue triangles and destination nodes are pink circles. The mobile nodes are the ones with black borders. Each node is presented in the location it occurred for the first time. The destination regions are marked as red dashed rectangles. A solid link depicts a space connection, i.e., one that occurs without message buffering (in the same time interval). Label 30 (24 s, 21 m) in Figure 14 indicates that the edge exists in slot number 30, the message has to be buffered for 24 slots in the relay before being forwarded, and that the space distance between the relay and the next hop node is 21 m.

#### 3.4.1. Gdańsk

**Figure 11:**
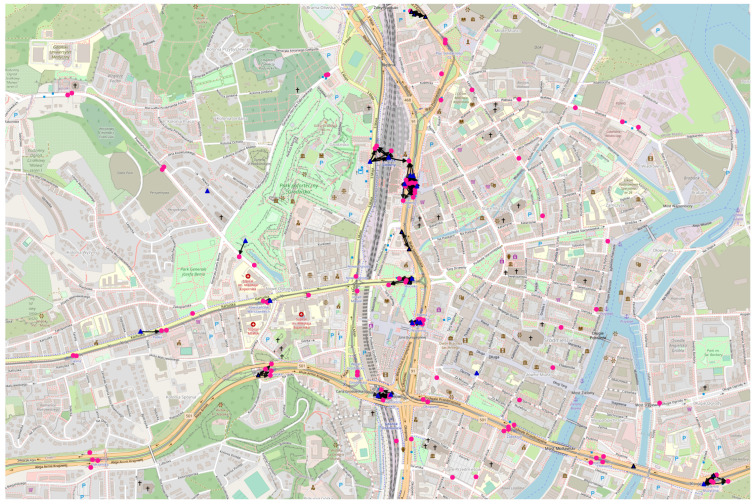
Example space connectivity graph in Gdańsk.

- Population:
  − Total: 486 thousand [35] in the metropolis, of around one million in northern Poland;
  − Density: 1797 per km^2^ [36];
- Public transport day routes: around 80 [37];
- Simulation area:
  − Latitude: 54.34398–54.36191;
  − Longitude: 18.62036–18.66666;
- Example space connectivity graph in Figure 11:
  − Slot length: 6 s;
  − Radio range: 100 m;
  − Nodes: 121—mobile relays: 9, stationary relays: 20, stationary destinations: 92;
  − Average node degree: 2.45, edges: 148, space cost: 6177 m, connected components: 66.

#### 3.4.2. Poznań

**Figure 12:**
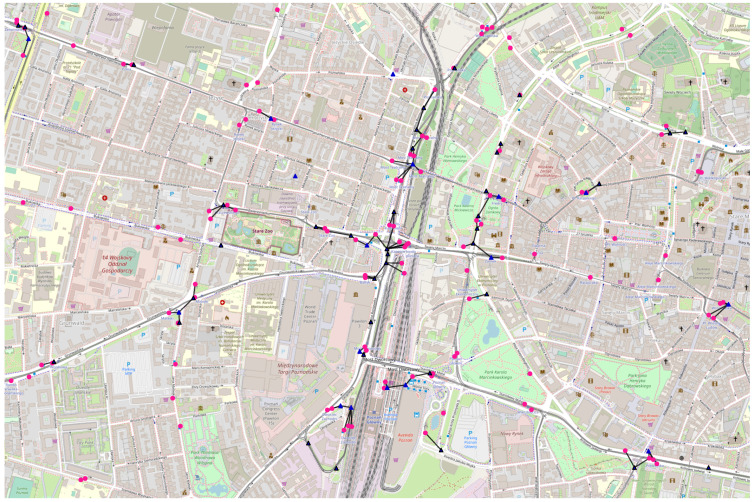
Example space minimum spanning forest in Poznań.

- Population:
  − Total: 547 thousand [38] in the metropolis, of almost one million in west-central Poland;
  − Density: 2031 per km^2^ [36];
- Public transport day routes: around 70 [39];
- Simulation area:
  − Latitude: 52.39853–52.41645;
  − Longitude: 16.88965–16.93389;
- Example space minimum spanning forest in Figure 12:
  − Time interval: 6 s;
  − Radio range: 100 m;
  − Nodes: 171—mobile relays: 38, stationary relays: 17, stationary destinations: 116;
  − Average node degree: 1.20, edges: 103, space cost: 4904.00 m, connected components: 68.

#### 3.4.3. Warsaw

**Figure 13:**
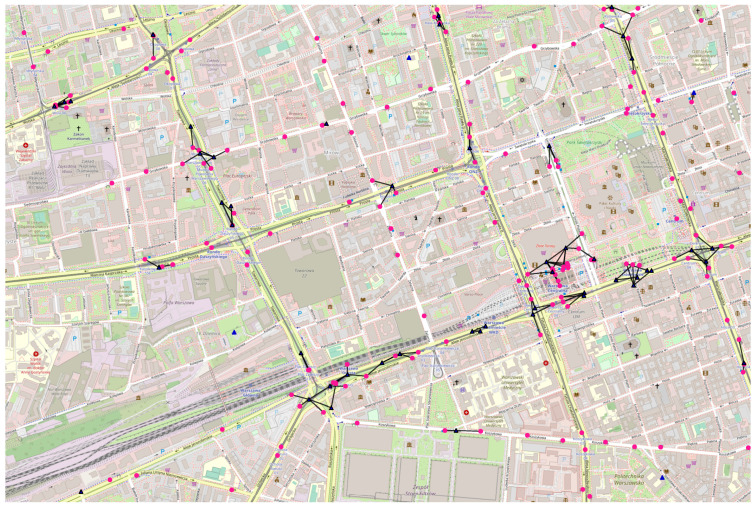
Example space maximum spanning forest in Warsaw.

- Population:
  − Total: 1.794 million [40] in the metropolis, of 3 million in east-central Poland;
  − Density: 3469 per km^2^ [36];
- Public transport day routes: around 190 [41];
- Simulation area:
  − Latitude: 52.22082–52.23879;
  − Longitude: 20.97058–21.01454;
- Example space maximum spanning forest in Figure 13:
  − Slot length: 6 s;
  − Radio range: 100 m;
  − Nodes: 213—mobile relays: 49, stationary relays: 4, stationary destinations: 160;
  − Average node degree: 1.10, edges: 117, space cost: 8199.00 m, connected components: 96.

#### 3.4.4. Wrocław

**Figure 14:**
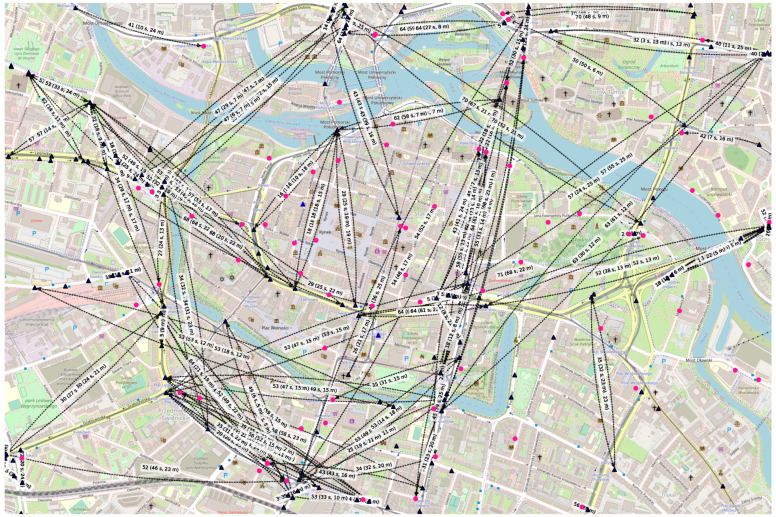
Example first-contact graph in Wrocław.

- Population:
  − Total: 674 thousand [42] in the metropolis, of around 1.25 million in southwestern Poland;
  − Density: 2192 per km^2^ [36];
- Public transport day routes: around 85 [43];
- Simulation area:
  − Latitude: 51.10015–51.11813;
  − Longitude: 17.01273–17.05570;
- Example first-contact graph in Figure 14:
  − Slot length: 6 s;
  − Radio range: 100 m;
  − Nodes: 217—mobile relays: 145, stationary relays: 2, stationary destinations: 70;
  − Average node degree: 1.66, edges: 180, space cost: 3089.00 m, connected components: 120.

### 3.5. Simulation Architecture and Parameters

**Figure 15:**
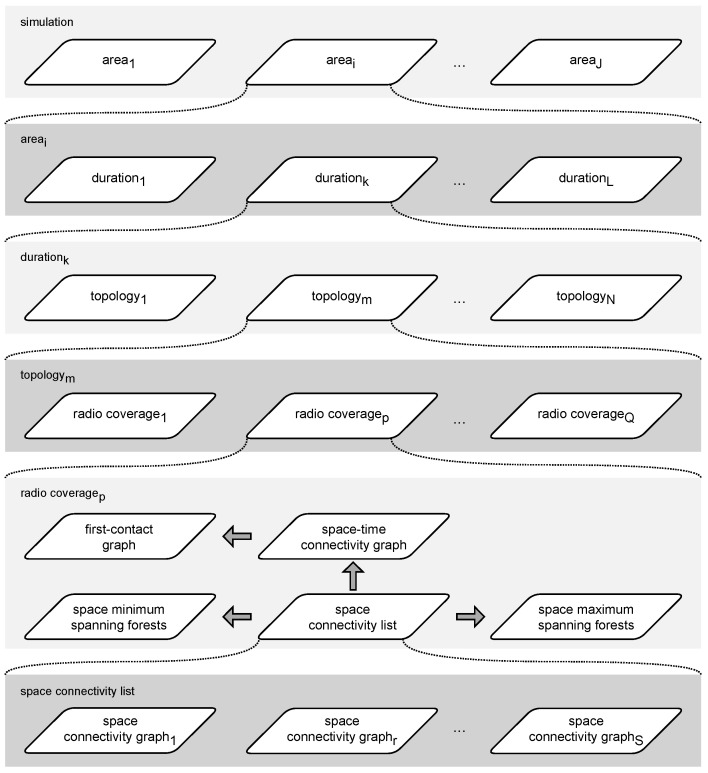
Simulation architecture.

To enable multi-faceted modeling and analysis, the simulation architecture is based on object-oriented data structures implemented as a hierarchy of nested lists, presented in Figure 15. The simulations are executed with the parameters related to the algorithms which are the main steps in the modeling flow depicted in Figure 10. The key simulation scope characteristics (numbers), denoted with single capital letters, resulting from the architecture and parameters are also indicated:

- Algorithm 1, *network device data to slots of space nodes* (NDD-SSN):
  − period: 27 November 2019 from 3:00 p.m. to 5:00 p.m.;
  − areas: 4 ⇒J=4;
    * $area_{1}$: ([54.34398,54.36191],[18.62036,18.66666])—Gdańsk;
    * $area_{2}$: ([52.39853,52.41645],[16.88965,16.93389])—Poznań;
    * $area_{2}$: ([52.22082,52.23879],[20.97058,21.01454])—Warsaw;
    * $area_{2}$: ([51.10015,51.11813],[17.01273,17.05570])—Wrocław;
  − topology lengths: (75, 150, 300, 600, 1200);
    * durations: (7.5 min, 15 min, 30 min, 60 min, 120 min) ⇒L=5;
    * topologies: (16, 8, 4, 2, 1) ⇒N=31;
  − slot length: 6 s ⇒S=1200;
  − classes: (mobile advanced, stationary simple, stationary advanced);
  − windows: (10 s, 24 h, 24 h);
  − relays: (mobile advanced, stationary advanced).
- Algorithm 2, *slots of space nodes to space connectivity list* (SSN-SCL):
  − radio coverage: omnidirectional;
    * radio ranges: (25 m, 50 m, 100 m) ⇒Q=3;
    * space distance: great-circle distance between two nodes.
- Algorithm 3, *space connectivity list to space–time connectivity graph* (SCL-STCG):
  − unit cost:
    * intra-slot time edge space unit cost: 0;
    * inter-slot time edge space unit cost: 0;
    * intra-slot space edge time unit cost: 0;
    * intra-slot time edge time unit cost: 0;
    * inter-slot time edge time unit cost: 1.

The node data sources for the simulation are grouped into three classes—mobile advanced, stationary simple, and stationary advanced, as introduced in Table 1. A data-lookup window is related with each of the classes. The widths of these were determined based on an analysis of the update frequencies of the sources. The data on mobile nodes are updated most frequently, i.e., as often as every few seconds. Therefore, a window of 10 s ensures that location changes will be reflected correctly in the modeled structures. Each node location is marked with the occurrence time. When the slot length is set to 6 s, a window of 10 s is also the means to correct brief node data or source outages—to avoid the node being missed in a single space connectivity graph when actually it was still present in the network. A longer window, in case of rapidly moving nodes and frequent data outages, may lead to misrepresentation of the node in its previous known location. Hence, connections may appear which, in reality, would not be possible to establish at that point in time since the node was, in fact, already at a different location. Also, the node could be able to establish links that were not modeled when the data were not available. Observation of the stationary nodes’ data leads to the conclusion that their location changes or is updated not more frequently than once every 24 h. Therefore, this interval is used as the window for fixed-node-related location data.

The simulations were conducted in four urban areas of interest based on the data gathered on Wednesday, 27 November 2019 between 3:00 p.m. and 5:00 p.m. This 2 h period was selected because it includes afternoon rush hours in the middle of a work week and enables coverage of all the desired modeling and analysis scenarios. In the modeling process, the period is divided in Algorithm 1 into topologies with durations defined by *topologyLength*. The values of interest are 75, 150, 300, 600, and 1200, which are the subset of a geometric sequence with a common ratio of 2. These are the numbers of space connectivity graphs in the space connectivity list of a given topology. They allow the modeling and study of network structures that are related, and yet, have different properties and time spans. Therefore, the trends connected to the matters of scalability and optimization can be investigated.

When the *slotLength* is set to 6 s, a series of topologies of 16, 8, 4, 2, and 1 space–time connectivity graphs are distinguished that last for 7.5, 15, 30, 60, and 120 min, respectively. The time of *slotLength* is expected to be sufficient to transmit the message between two neighboring nodes. Unlimited message storage (buffer) is assumed in each relay. Omnidirectional radio coverage is modeled for three effective radio ranges, i.e., 25 m, 50 m, and 100 m. These range limits are based on empirical observations that current popular sensing-related short-range wireless connectivity technologies tend to provide up to around 100 m range at higher throughputs in outdoor urban non-line-of-sight scenarios, depending on the transmit power [44] and data transmission parameters [45]. The space distance between two nodes is determined with the haversine formula, which computes the great-circle distance between two points on a sphere [12]. For each of the resulting graphs, minimum and maximum spanning forests are constructed.

Then, space–time connectivity graphs (networks) are modeled based on each of 31 space connectivity lists. The default values of the unit costs of Algorithm 3, SCL-STCG, are used. By this means, correct time-shortest paths can be determined using Dijkstra’s algorithm based on the time distance weight of the edges. The process set up in this way aims to balance the buffering time with message forwarding, and therefore, buffering resources use with transmission-related power consumption of the relays. The related first-contact graphs are constructed as well. Time distance in those graphs means how much time, i.e., time slots, has to pass before the node will be close enough to the neighboring node (device) to establish a connection.

The chosen simulation scope and parameters resulted in a total of 43,944 graphs being modeled, as listed in Table 2.

### 3.6. Simulation Study Metrics

The studied metrics are depicted in the next sections, first in absolute values, then some are presented as ratios (percentages) related to the reference ones. The metrics are listed in this section in relation to the first type of modeled structures they are discussed for. Other types may use the same or related metrics. The parameters are further divided into those that pertain to nodes (devices) and those that relate to edges (connections) of the graphs (networks). All of the used metrics are non-negative.

1. Space connectivity:
  (a) nodes:
    - stationary destination nodes—the number of stationary destinations;
    - stationary relay nodes—the number of stationary relays;
    - mobile relay nodes—the number of mobile relays;
    - mobile relay nodes to all nodes ratio—the percentage of mobile relay nodes as compared to the number of all nodes;
    - all nodes—total number of nodes;
    - connected components—the number of sets of nodes that are connected with each other by direct or indirect paths;
    - nodes per component—average number of nodes in a component;
  (b) edges:
    - average node degree—average number of edges adjacent to a node;
    - edges—total number of edges;
    - cost—the sum of space distances of all edges;
2. Space–time connectivity:
  (a) nodes
    - instances per node—the number of time nodes (instances) per unique device (space node)
    - instances per mobile relay node—the number of time nodes (instances) per unique mobile relay device (space node).

## 4. Space Connectivity Analysis

Following the objectives and parameters defined in Section 3, Algorithm 1, *network device data to slots of space nodes* (NDD-SSN) was used to select and group physical device (space nodes) data into time slots. Then, Algorithm 2, *slots of space nodes to space connectivity list* (SSN-SCL) was applied to construct *space connectivity lists* (SCLs) related to each city and modeled radio ranges. With 6 s slots and a 2 h period of interest, each SCL consists of 1200 subsequent *space connectivity graphs* (SCGs). Four cities and three radio ranges require twelve SCLs. This results in 14,400.00 space connectivity graphs with 2.3 million nodes and 1.6 million edges in total. Those space connectivity lists are both the object of the analysis in this section, as well as the key starting elements to construct the space–time connectivity graphs modeled in the next sections.

### 4.1. Space Connectivity Nodes

**Figure 16:**
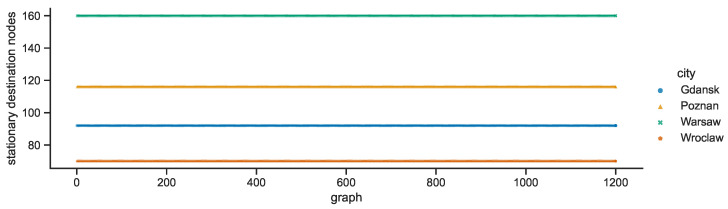
Stationary destination nodes in space connectivity graphs.

**Figure 17:**
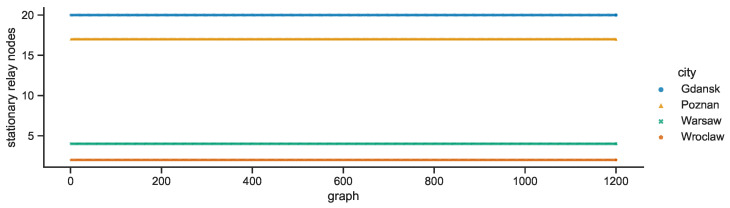
Stationary relay nodes in space connectivity graphs.

Each city network is characterized by a constant number of stationary destination nodes—Warsaw: 160, Poznań: 116, Gdańsk: 92, Wrocław: 70—as compared in Figure 16. The numbers of stationary relay nodes presented in Figure 17 are also constant but the decreasing order is quite different—Gdańsk: 20, Poznań: 17, Warsaw: 4, Wrocław: 2.

**Figure 18:**
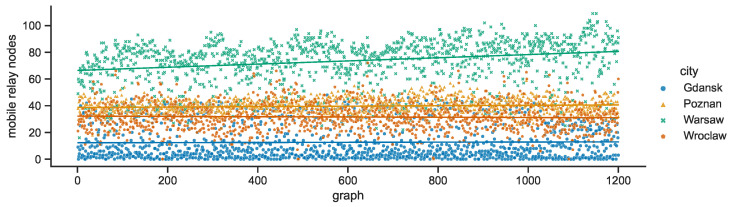
Mobile relay nodes in space connectivity graphs.

The mobile relay numbers are the ones that vary significantly across cities; they can be examined in Figure 18. These numbers also change over time in the areas of interest, although they oscillate around characteristic values—Warsaw: 74, Poznań: 39, Wrocław: 32, Gdańsk: 13. Only in the case of Warsaw can a distinctive slope increase in the regression line be observed. This shows that the number of mobile relays (vehicles) increases on average, which may be caused both by the need to address the increasing number of rush hours commuters, and by the traffic jams that may slow down the nodes. Warsaw also expresses the largest deviations from the regression line, especially for lower values. Conversely, in Gdańsk the numbers increase more dynamically but the maximum values, i.e., the number of public transport vehicles, drop by around 10 in about the last 150 graphs (15 min). The number of mobile relay nodes in Wrocław tends to be more evenly distributed while in Poznań the numbers are more tightly grouped.

**Figure 19:**
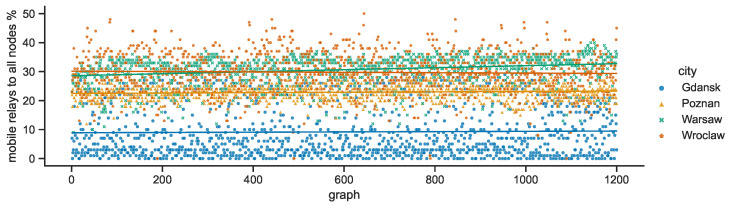
Ratio of mobile relay nodes to all nodes in space connectivity graphs.

**Figure 20:**
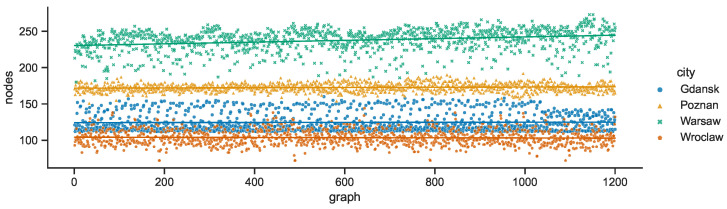
All nodes in space connectivity graphs.

The number of mobile nodes represents a different percentage of all the nodes in given city. What is visible in Figure 19 is that the relative numbers are substantially lower in Gdańsk, while Warsaw and Wrocław tend to be close and characterized by the highest ratios, with Poznań falling slightly behind—Warsaw: 30, Wrocław: 30, Poznań: 23, Gdańsk: 9. The sums of the respective stationary node numbers are the lower bounds of the total numbers of nodes in SCL compared in Figure 20. The deviations, and hence the maximum values, are related to the number of mobile relay nodes changing over time—Warsaw: 237, Poznań: 172, Gdańsk: 125, Wrocław: 104.

### 4.2. Space Connectivity Edges

**Figure 21:**
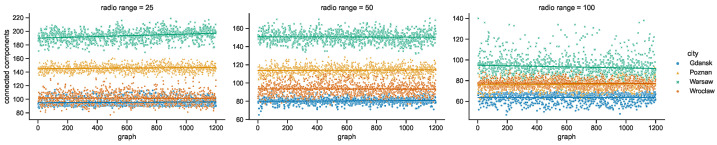
Connected components in space connectivity graphs.

**Figure 22:**
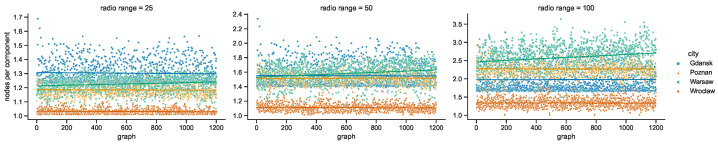
Average number of nodes per connected component in space connectivity graphs.

**Figure 23:**
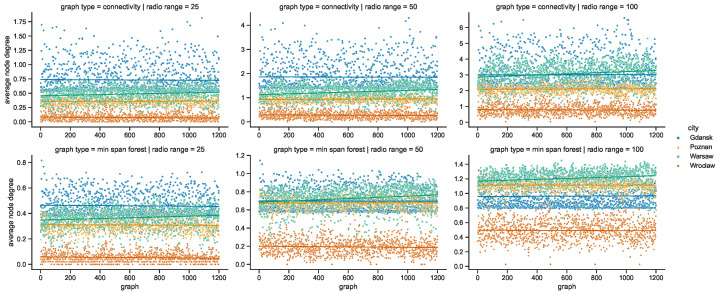
Average node degree of space connectivity graphs and minimum spanning forests.

**Figure 24:**
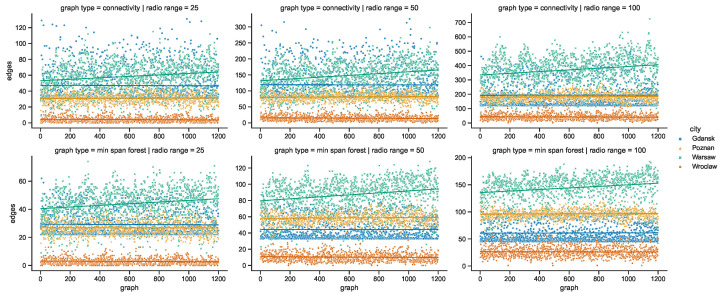
Edges of space connectivity graphs and minimum spanning forests.

While the numbers of nodes are shared by the networks in the given areas of all of the investigated radio ranges, other graph metrics vary. It is clearly visible in the graphs of Figure 21 that as the radio range increases, the number of connected components decreases. The topologies in Wrocław are the most sparse, while in other cities the average number of nodes per connected component depends more on the radio range, as compared in Figure 22. The number of average node degrees in Figure 23 and number of edges in Figure 24 increase with radio range. This follows the intuitive understanding that with increasing radio range, the resulting network will be less fragmented. Moreover, in different cities the changes in node radio range influence the structure of the network to different extents. This is related to the unique infrastructure topology and mobility patterns of each area. It is visible in particular when the changes in Warsaw and Wrocław are compared. Also, the average node degrees and edge numbers suggest that nodes in Poznań and Warsaw are denser and evenly distributed.

**Figure 25:**
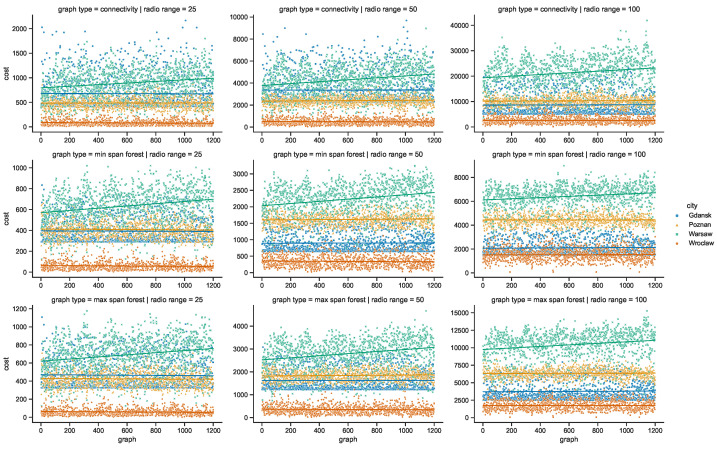
Cost of space connectivity graphs and spanning forests.

Moreover, the parameters of minimum spanning forests constructed in space connectivity graphs are influenced by increasing radio range in different way in different cities. When the networks are less fragmented, the number of edges in such trees, as well as the average node degrees, increase. Albeit, they do not influence the distributions in the same way, as visible in the bottom row graphs of Figure 23 and Figure 24. Those figures do not present the parameters of maximum spanning forests since the numbers of edges and average node numbers were the same as for minimum spanning forests. The costs of the structures are different though, as presented side-by-side in Figure 25. There, the larger the radio range, the greater the cost and the difference between the costs of the minimum and maximum spanning forests.

### 4.3. Space Connectivity Relationships

**Figure 26:**
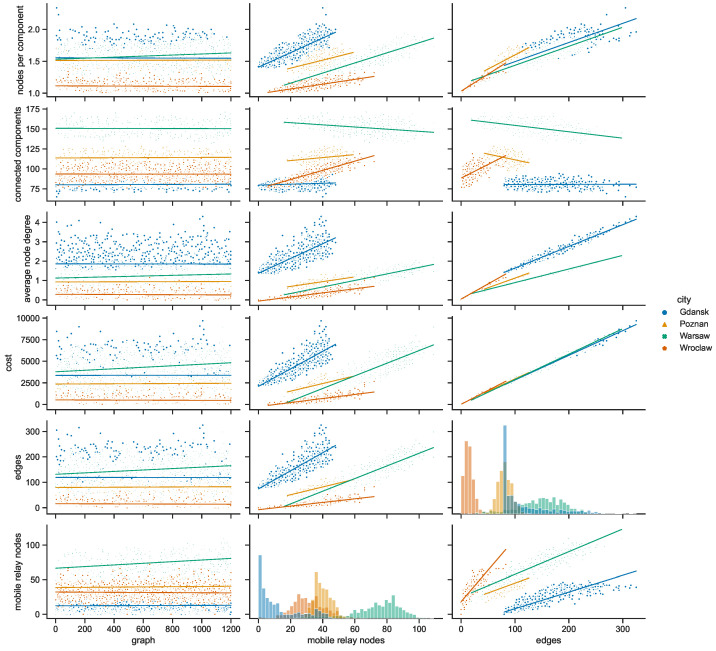
Space connectivity parameters in Polish cities at 50 m radio range.

Figure 26 presents the relationships between multiple variables of the space connectivity graphs when the radio range was set to 50 m. It has already been shown that each range results in unique topological features of the networks. Although, this particular range, being the middle one of the simulated values, was selected to give an overview of the general differences in characteristics of the urban areas (cities) under scrutiny. One set of parameters, i.e., the columns, changing along the horizontal axis of the figure, covers the slot (graph) number and the number of mobile relay nodes, as well as the number of edges. The other set, the rows, changing along the vertical axis of the figure, covers the numbers of mobile relay nodes, the numbers of edges, graph costs, average node degrees, and the number of connected components.

Both presented histograms, i.e., the number of mobile relay nodes and the number of edges, suggest that each urban area and infrastructure has its own features and parameters. Almost every other related graph proves this even more strongly by presenting mostly disjoint groups of measurements for each city. They display, though, a degree of correlation in terms of the increasing trends they follow. Interestingly, it is only in Warsaw that the number of connected components clearly decreases with the increase in the number of mobile relay nodes and edges. In Wrocław, the increases in the number of mobile relay nodes and in the number of edges are followed by an increase in the connected components. At first glance, one could think that this means that despite becoming more populated with mobile relays, the structure becomes more disconnected. Here, the situation is quite different, and when more mobile relay nodes are present, more nodes can be connected, and yet they form new disconnected components rather than becoming connected to the larger ones. The conditions in Poznań may seem even less intuitive, with the number of connected components increasing with the number of mobile relays and decreasing with the increase in the number of edges. This means that with more graph edges, the nodes are, on average, connected in larger groups (components). In this urban area, more mobile relays, like in Wrocław, cause more nodes to be connected and constitute more smaller groups. In Gdańsk, which is the city with the lowest number of mobile relay nodes, those numbers and slope features are influenced only to a small extent, but with noticeable deviations from the trend. The highest direct correlation and overlapping is present between the number of edges and the costs of the trees in all of the cities. The relationship is almost linear, with little deviation, which suggests that the average edge costs are similar. The average node degrees differ more, and yet, depend on the number of mobile relays in a way resembling the relationship between the number of mobile relay nodes and edges. In terms of edges to average node degree relationship, the city-related distributions appear as distinctive linearly condensed groups.

To investigate the features in more detail, the space connectivity parameters of each city are presented and analyzed for all radio ranges separately in the next subsections.

#### 4.3.1. Space Connectivity in Gdańsk

**Figure 27:**
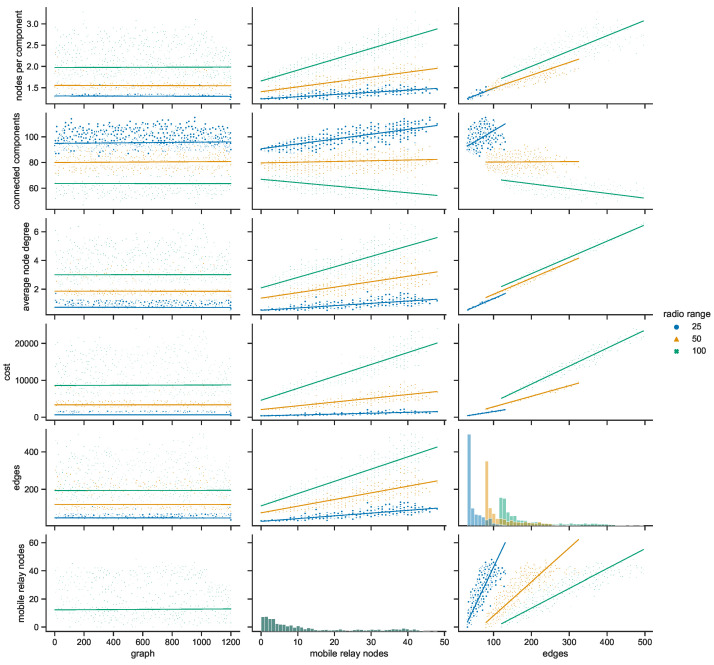
Space connectivity parameters in Gdańsk.

Taking a closer at the parameters of the space connectivity graphs in Gdańsk in Figure 27 reveals that the trends related to the connected components are linked not only with the number of mobile relay nodes and edges but also with the radio range. When the range increases from 25 to 50 m, the slope of the regression line changes from increasing to almost horizontal; when the radio range extends as far as 100 m it begins to decrease. This tendency is also influenced by the largest number of stationary relays of all studied cities, as visible in Figure 17. This means that with more radio range more nodes could be reached and connected to make larger components of the graph. The average node degrees, and hence, complexity and costs, of such structures are significantly higher. It cannot be overlooked that in Gdańsk usually only a few mobile relays were present, which is about one order of magnitude less than in other cities. The distribution of the numbers of edges is correlated with the distribution of mobile relay nodes, becoming more flattened and shifted in the direction of higher values as the radio range increases. It should be noted that a similar situation occurs in the relationship between the number of edges and average node degree.

#### 4.3.2. Space Connectivity in Poznań

**Figure 28:**
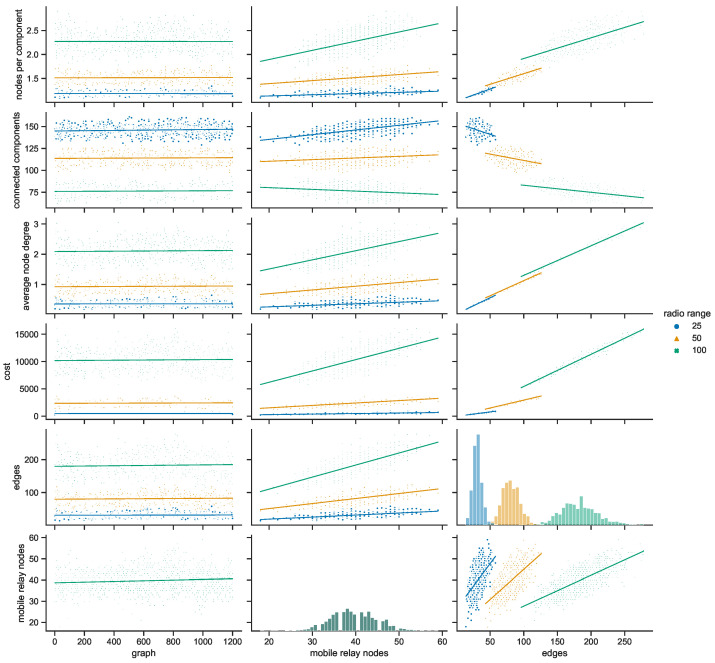
Space connectivity parameters in Poznań.

The space connectivity parameters in the networks modeled in Poznań and compared in Figure 28 at first seem similar to the ones in Gdańsk. They share common features, but a number of the characteristics are quite distinctive. The first difference is the much larger number of mobile relays in Poznań. The distributions of edge numbers are more Gaussian and shifted as the radio range increases. The average node degrees and graph costs are comparable in nature to Gdańsk. The slope of the trend of connected components in relation to edges is decreasing in all the range cases. This shows that, even at the lowest radio range, the numbers of mobile relays are high enough and their routes coincide with other nodes at a level which makes the network more connected and dense. The situation is also influenced by seventeen stationary relays, as introduced in Figure 17. It can be said that Poznań is the city with a topology which is the easiest one in which to achieve a high level of connectivity at the lowest cost.

#### 4.3.3. Space Connectivity in Warsaw

**Figure 29:**
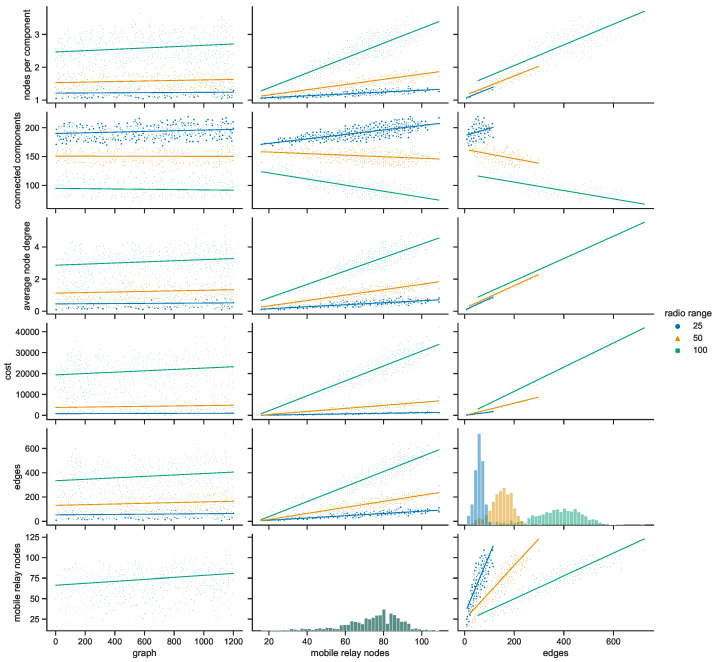
Space connectivity parameters in Warsaw.

The average number of mobile relay nodes in Warsaw is almost six times higher than in Gdańsk. Although, the distributions of the metrics of the Warsaw space connectivity graphs in Figure 29 share more similarities with Gdańsk than with Poznań. In Warsaw, the numbers of connected components, edges, and graph costs are twice as high as the ones in Gdańsk. Moreover, most of the metrics increase over time. The average node degree is the metric that stays at a similar level in both cities, being more compressed in Warsaw. Importantly, despite having the largest number of mobile relays, for each radio range, there is a number of graphs with only a few edges. Hence, stationary network nodes are more distributed and disconnected on their own than in Gdańsk and Poznań. Only four stationary relay nodes (see Figure 17) do not improve the connectivity enough. Although, the isolated stationary nodes become connected owing to the largest, and increasing over time, number of mobile relay nodes. This results in the highest numbers of edges of all the cities, as well as the largest graph costs.

#### 4.3.4. Space Connectivity in Wrocław

**Figure 30:**
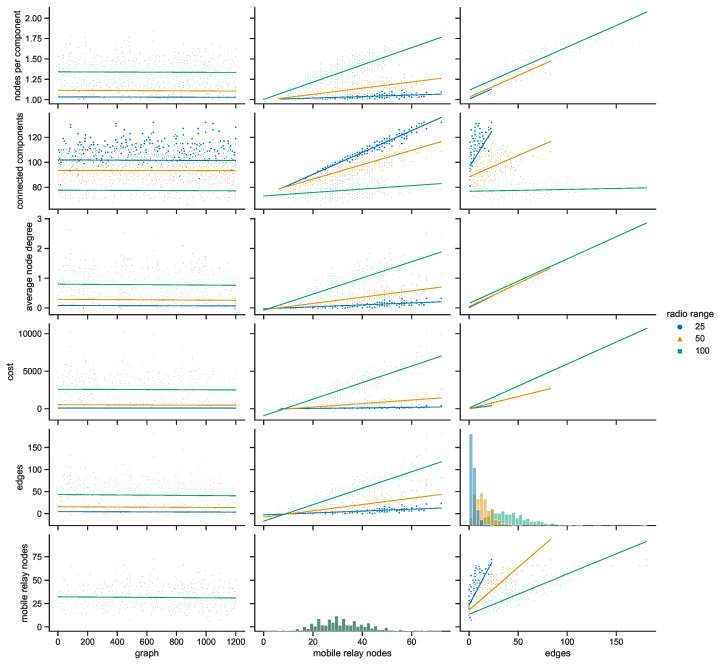
Space connectivity parameters in Wrocław.

In Figure 30, it is striking that Wrocław is the only city in which, in spite of tens of mobile relays, there are numerous graphs with no edges. This means that stationary nodes are heavily disconnected, especially when the radio range is at its lowest. Moreover, many of them are located beyond the routes and range of mobile relays. Wrocław is also the city with the lowest number of stationary relays, with only two nodes of this kind (see Figure 17). On the one hand, despite Wrocław being the city with the lowest overall numbers of nodes, it has around 30% of mobile relays, on a par with Warsaw. This is the largest percentage among the cities under scrutiny, as presented in Figure 19. On the other hand, the exact number of mobile relay nodes is comparable to Poznań, which has quite different distributions related to the connected components. In the edges to connected components relation, the trend is highly increasing at lower radio ranges, becoming almost vertical for the largest range. This stresses the uniqueness of both the topology of the area and the networked infrastructure deployment.

## 5. Space–Time Connectivity Analysis

Based on the space connectivity graphs analyzed in Section 4, *space–time connectivity graphs* (STCGs) were constructed using Algorithm 3, *space connectivity list to space–time connectivity graph* (SCL-STCG). Then, each STCG was used to build the related *first-contact graph* (FCG) with Algorithm 4, *space–time connectivity graph to first-contact graph* (STCG-FCG). While an STCG, which is a *time-expanded graph*, is mostly an intermediate network modeling structure, an FCG is a *time-aggregated graph* with a more practical meaning. The analysis of the FCG provides a general and easier to comprehend impression of how the space–time network changes in real environment and how the adjacencies occur for the first time. Therefore, the results for 372 graphs of both space–time types are presented and discussed side by side.

**Figure 31:**
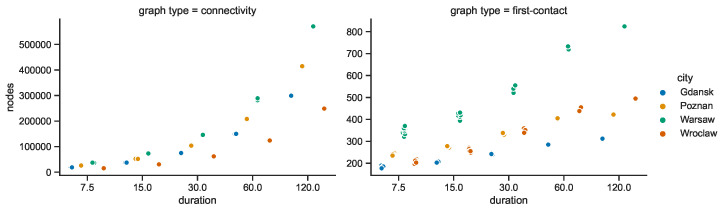
All nodes in space–time connectivity and first-contact graphs.

The graphs were constructed in four cities, at three radio ranges, and for five durations that divide the period of interest into respective networks. Each consecutive duration is twice the preceding one. Therefore, due to this geometric growth nature, the increasing trend visible in Figure 31 for the connectivity graph which seems exponential is in fact of a rather linear nature. Conversely, it looks linear in the case of the first-contact graph, and hence, it is of a more logarithmic type.

**Figure 32:**
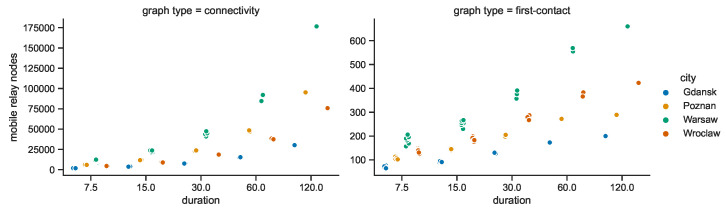
Mobile relay nodes in space–time connectivity and first-contact graphs.

Since the numbers of stationary nodes were constant in the space connectivity graphs, in the respective space–time connectivity graphs they also remain at the same level in each area and duration. The numbers of mobile relay nodes presented in Figure 32 are varying, resulting in correlated changes in the overall number of nodes in the graphs. This is caused by the fact that not only the number of mobile relays changes over time but also the number of mobile nodes (the devices) present in the area changes. Moreover, some leave the area while others enter at different points in time. In the spatiotemporal network there are more unique nodes (devices) than in each single space connectivity graph. This is the reason why the number of mobile relays in first-contact graphs are at least a few times higher than in the space connectivity graphs in Figure 18. This number hardly exceeds 200 mobile relay nodes in Warsaw for the shortest duration. For the longest one, the number reaches 660 mobile relays. In other areas the numbers are lower. Interestingly, Poznań-related STCGs consist of more mobile nodes than the ones related to Wrocław. In FCGs, due to the structure of the connections, the situation is the opposite. Gdańsk remains the area with the lowest number of mobile relay nodes for all durations.

**Figure 33:**
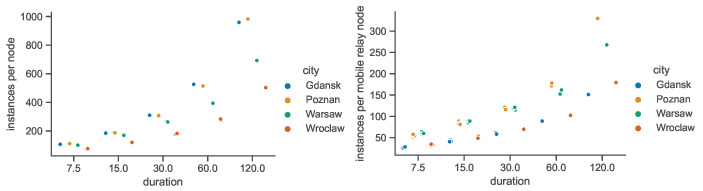
Instances per node in space–time connectivity graphs.

Importantly, the STCG construction algorithm is the reason for large numbers of nodes being present in space–time connectivity graphs, since each node of each SCG is represented by two instances in the STCG. This means that for each unique device (space node) in the observation area and duration, multiple instances (time nodes) are present in the space–time connectivity graph, as depicted in Figure 33. For example, the number of nodes for the 30 min duration in the STCGs ranges from around 75 thousand for Gdańsk to more than 140 thousand for Warsaw. These correspond to around 240 nodes for Gdańsk and 540 nodes for Warsaw in the related first-contact graphs. The numbers of instances of mobile relays are the highest in Poznań and Wrocław. This means that in those areas of interest particular mobile relays are on average present for the longest total time. Figure 33 shows the numbers only for the space–time connectivity graphs because in the first-connectivity graphs each actual device is represented by a single node.

**Figure 34:**
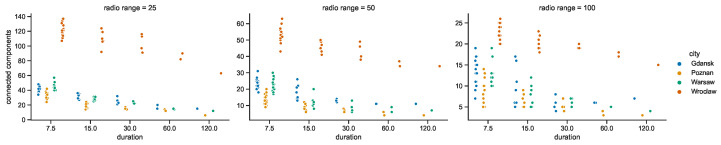
Connected components in first-contact graphs.

In terms of the connected components in the first-contact graphs, see Figure 34, their numbers for Gdańsk, Poznań, and Warsaw are lower than they are on average in the space connectivity graphs compared in Figure 21. This difference grows when the network duration and radio ranges increase. The same trend can be observed in Wrocław but there is a difference. At the shortest durations and the smallest radio range, the numbers of connected components exceed the ones in the SCGs. This shows that the infrastructure in Wrocław is more fragmented and more time is needed to make the network more connected.

**Figure 35:**
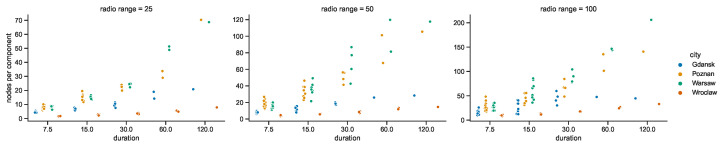
Nodes per connected component in first-contact graphs.

**Figure 36:**
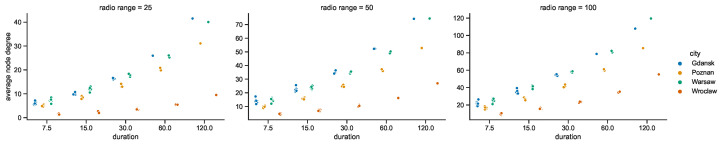
Average node degree in space–time first-contact graphs.

The opposite trends can be observed in relation to the numbers of nodes per component in Figure 35 and the average node degrees in Figure 36, when compared to Figure 22 and Figure 23, respectively, which are related to these parameters of the space connectivity graphs. The much higher values of the space–time metrics are of key significance, because they prove that when mobile relay nodes are used to build a space–time network, the momentarily (temporarily) disconnected components (parts of the network) are connected over time and, as a result, larger and more dense time-spanning networks are constructed. Wrocław falls behind considerably, but it needs to be taken into account that the underlying space connectivity graphs were of the lowest average node degree as well.

## 6. Summary

This paper introduces new network connectivity modeling algorithms designed for realistic heterogeneous urban sensor networks. The presented methods use emerging publicly available data sources which provide the locations of different elements of urban infrastructure, public transportation vehicles, etc. Other types of related information are usually available as well. The family of related algorithms is presented as a set of commented pseudocodes, examples, and clarifications. A multidimensional simulation architecture has been proposed and used to construct static (momentary) and dynamic (time-changing) topologies, i.e., space connectivity graphs, space–time connectivity graphs, and first-contact graphs, in selected areas of four large Polish cities—Gdańsk, Poznań, Warsaw, and Wrocław.

Key observations related to space connectivity analysis:

- Large-scale network modeling with *space connectivity graphs* (SCGs) and multidimensional analysis can be conducted based on open data;
- The unique topology and infrastructure features of each urban area influence the networks that are constructed;
- When conducting analyses of the data obtained for individual cities, it can be seen that the highest overall number of nodes does not necessarily correspond to the population density of the individual cities.
- Mobile relays are present in the areas of interest in varying numbers and with varying distributions, which depend on the infrastructure of the cities concerned, including the number of daily public transport lines, vehicle frequency, street topology, and density of stops;
- Changing the radio range of the nodes affects the modeled networks differently, depending on the distribution of the nodes. Immediate topologies are more fragmented for smaller radio ranges. In more fragmented areas, increased radio coverage is required to connect more nodes;
- The radio coverage affects the costs of space connectivity graphs and their associated minimum and maximum spanning forests. Studies have shown that as radio coverage increases, the number of edges and the cost of structures also increase. These increases are exponential, so the radio parameters of the designed networks must be carefully planned. In this way, excessive use of the wireless medium, as well as the computing and storage resources of the nodes, can be avoided;

Key observations related to space–time connectivity analysis:

- *Space–time connectivity graphs* (STCGs), which consist of as many as hundreds of thousands of nodes, can be modeled based on *space connectivity lists* (SCLs) and used as the intermediate structure in space–time network modeling;
- *First-contact graphs* (FCGs) are valuable compact-form indicators that capture how space–time networks develop over time and how the adjacencies occur for the first time;
- The structures become more complex when network duration and radio coverage grows. This means that topology and connectivity increase as well;
- The sets of mobile nodes present in the areas change over time—some enter and some leave at different moments. In a space–time connectivity graph, there are more unique space nodes (individual devices) than in a single space connectivity graph. As a result, the number of mobile relays in first-contact graphs is at least a few times higher than in the related space connectivity graphs;
- The average node degrees and the number of connected components are much higher in first-contact graphs than they are in space connectivity graphs. This proves that using mobile relays to construct space–time networks enables the momentarily disconnected parts of a network to be connected over time. As a result, larger and denser time-spanning topologies are constructed.

Main contributions of presented work:

- Urban sensor network graph-based modeling algorithms:
  − space connectivity modeling;
  − space–time connectivity modeling;
  − first-contact modeling;
- Simulation study of introduced models and algorithms:
  − methodology for multidimensional modeling and analysis;
  − simulation architecture and custom-developed environment;
  − comparative investigation and observations for four Polish cities.

Suggested directions for further research:

- Urban sensor network modeling:
  − Development of a reliable parametric network topology generator based on long-term observations of open data and timetables;
  − Construction of movement traces based on gathered node location open data;
  − Use of introduced modeling architecture as an element of stationary relay deployment planning;
  − Use of presented modeling algorithms in other fields:
    * social trends analysis;
    * multi-criteria route optimization for public service vehicles;
    * urban planning of bicycle paths, street infrastructure, etc.;
- Graph-based study of presented algorithms:
  − Investigation of extended scale and scope:
    * more data sources, including the closed ones, when available;
    * more areas of different locations and sizes, e.g., suburbs, small cities, countrysides, etc.;
  − Advanced radio connectivity modeling:
    * use of actual connectivity data, when available;
    * use of complex radio coverage models.

In total, nearly 44 thousand graphs were built and enabled the study of the modeled networks. Both well-known and newly introduced graph-theory metrics were presented. The key features, trends, and relationships were analyzed, compared, and discussed, showing the usability of the introduced urban sensor network modeling algorithms. Further research directions were presented.

## Figures and Tables

**Table 1:** Urban open data sources used in simulation study.

City	Class	Scope	Format	Updates	Provider
Gdańsk	Mobile advanced	Buses and trams [23]	JSON	20 s	Open Gdańsk
	Stationary simple	Public transport stops [24]	JSON	24 h	Open Gdańsk
	Stationary advanced	Ticket machines [25]	JSON	24 h	Open Gdańsk
Poznań	Mobile advanced	Buses and trams [26]	protobuf	Continuous	ZTM Poznań
	Stationary simple	Public transport stops [27]	JSON	Infrequent	Poznan City Hall
	Stationary advanced	Ticket machines [28]	JSON	Infrequent	Poznan City Hall
Warsaw	Mobile advanced	Buses and trams [29]	JSON	10 s	City of Warsaw
	Stationary simple	Public transport stops [30]	JSON	Infrequent	City of Warsaw
Wrocław	Mobile advanced	Buses and trams [31]	JSON	Continuous	Open Data Wrocław
	Stationary simple	City bike rental stations [32]	JSON	5 min	Open Data Wrocław
	Stationary simple	Vozilla parking lots [33]	JSON	Continuous	Open Data Wrocław
All	Stationary advanced	Air quality meters [34]	JSON	Continuous	Airly

**Table 2:** Numbers of modeled graphs.

Category	Type	Number of Graphs
Space	Connectivity	14,400
	Minimum spanning forest	14,400
	Maximum spanning forest	14,400
Space–time	Connectivity	372
	First-contact	372
In total		43,944

## Data Availability

Data are contained within the article.
